# Synthesis of Cleavable Polyolefins via Ring-Opening Insertion Metathesis Polymerization (ROIMP) of Cyclopentene with Unsaturated Polyester Oligomers

**DOI:** 10.1021/acs.macromol.5c03036

**Published:** 2025-12-22

**Authors:** Vajk Farkas, Ádám Erdélyi, Kristóf Varga, Dang Vu Hai, Momoko Ishii, Márton Nagyházi, Gábor Turczel, Pooja Dubey, László Trif, Ole Osterthun, Paul T. Anastas, Jürgen Klankermayer, János Moczó, Róbert Tuba

**Affiliations:** † Institute of Materials and Environmental Chemistry, 280964 Research Centre for Natural Sciences, Magyar tudósok körútja 2, Budapest 1117, Hungary; ‡ Department of Organic Chemistry and Technology, 61810 Budapest University of Technology and Economics, Szent Gellért tér 4., Budapest 1111, Hungary; § Yale Center for Green Chemistry and Engineering, 5755 Yale University, New Haven, Connecticut 06511, United States; ∥ Institut für Technische und Makromolekulare Chemie, RWTH Aachen University, Worringerweg 2, Aachen 52074, Germany; ⊥ Research Centre for Biochemical, Environmental and Chemical Engineering, Department of MOL Hydrocarbon and Coal Processing, University of Pannonia, Egyetem u. 10, Veszprém 8210, Hungary

## Abstract

The replacement of persistent plastics with chemically recyclable and environmentally benign alternatives is an urgent environmental challenge. Although an increasing number of degradable polymers are now available, many face limitations that prevent their efficient direct substitution for durable plastics, such as polyethylene. Here, we present a methodology for the synthesis of polyolefin copolymers containing cleavable units via Ring-Opening Insertion Metathesis Polymerization (ROIMP) of cyclopentene (**CP**) with unsaturated polyester and polycarbonate oligomers. In the first step, diallyl ester oligomers were prepared through acyclic diene metathesis (ADMET) polymerization followed by copolymerization with **CP** via ROIMP to yield long-chain polypentenamer (**PPe**) dyads separated by singular, easily cleavable ester or carbonate functionalities. Hydrolysis of the resulting cleavable **PPe** elastomers produced low-molecular-weight, telechelic OH-end-functionalized **PPe** oligomers. Subsequent hydrogenation using Wilkinson’s catalyst afforded saturated long-chain hydrocarbon polymers with randomly distributed cleavable subunits. The scalability of this approach was demonstrated for the ROIMP of **CP** with oligo-diallyl succinate (*oligo*-**DAS**).

## Graphical abstract

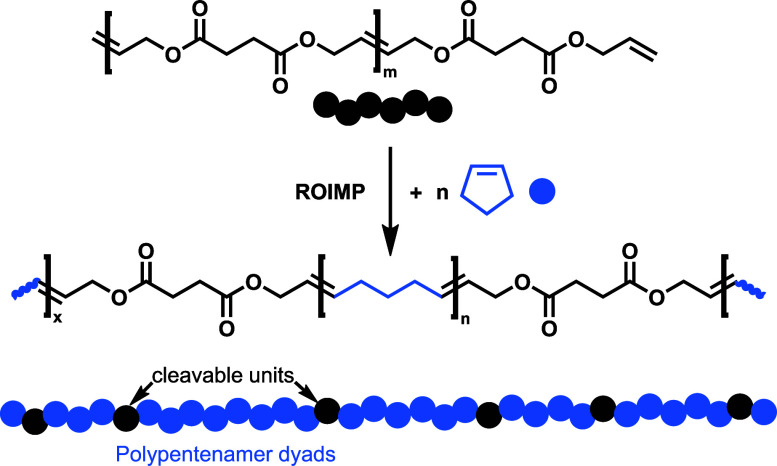

## 1 Introduction

Plastic pollution is one of the most urgent environmental challenges facing the modern world. Polyolefins (**POs**), the most widely produced plastics, are primarily synthesized from fossil-derived alkenes through energy-intensive cracking processes.^1,2^ While **POs** are indispensable in packaging and consumer applications, they exhibit poor environmental compatibility, and their chemical recycling processes remain inefficient and energy intensive. As a consequence, their accumulation in the environment has emerged as a significant concern, primarily owing to their persistence and substantial contribution to microplastic pollution.^3^

Polypentenamer (**PPe**)—polyalkenamer, a special type of polyolefins—holds particular significance among synthetic rubbers due to its physical properties closely resembling those of natural rubber.^4^ **PPe** gained attention for their potential^5,6^ as specialty elastomers in automotive components and consumer goods, in high-impact polymer compositions. Another typical polyolefin example is the polyethylene (**PE**) whose production grows at an annual rate of 3.7%, reaching 110 million tons in 2022.^7,8^ Most **PE** products, especially single-use packaging, are discarded within 1 year, with the majority ending up in landfills or incinerators. Polyolefins represent highly persistent plastics, with environmental half-lives that may extend to several hundred years. These limitations underscore the necessity for more broadly sustainable alternatives with reduced environmental impact.

Polyester-based, cleavable, and biodegradable polymers such as poly­(butylene succinate) (**PBS**), poly­(butylene adipate-*co*-terephthalate) (**PBAT**), and poly­(butylene carbonate) (**PBC**) exhibit promising material properties comparable to those of polypropylene (**PP**) and low-density polyethylene (**LDPE**) (Scheme 1).^9–11^

**Scheme 1:**
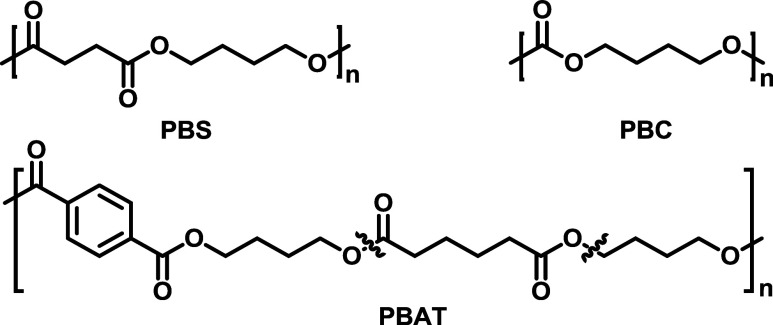
Chemical Structures of Poly­(butylene succinate) (**PBS**), Poly­(butylene carbonate) (**PBC**), and Poly­(butylene adipate-*co*-terephthalate) (**PBAT)**

In our laboratory, we have developed efficient catalytic methodologies, including a ruthenium-catalyzed isomerization–metathesis (ISOMET) process that enables the transformation of polyethylene (PE) waste and methyl oleate into propylene^12–14^ —a key intermediate for the synthesis of allyl alcohol and 1,4-butanediol.^15^ Similarly, we have demonstrated the conversion of polyunsaturated fatty acids into 1,6-hexanediol a precursor of adipic acid, a monomer utilized in the production of **PBAT**.^16^ Furthermore, the synthesis of terephthalic acid from lignin-derived feedstocks has also been reported.^17^

Building on these more eco-friendly pathways, we explored the use of diallyl esters derived from plastic waste^14,15^ or renewable feedstocks^16^ for advanced polymer design. Through ADMET oligomerization followed by Ring-Opening Insertion Metathesis Polymerization (ROIMP)^18–20^ with cyclopentene (**CP**), we create cleavable polyolefin copolymers incorporating hydrolyzable ester or carbonate units.^16,20–26^ It has recently been reported that **CP** can be synthesized not only from petrochemical feedstocks but also from renewable materials using hybrid bio- and chemocatalytic methods.^27^ These materials are expected to exhibit enhanced environmental compatibility, offering advanced polymer design strategies toward the development of next-generation circular plastics.^28–31^

## 2 Results and Discussion

### 2.1 Acyclic Diene Metathesis (ADMET) Polymerization of Diallyl Esters

Diallyl esters have been reported to exhibit low activity in ADMET polymerization when using either Grubbs^32^ or Schrock^33,34^ catalysts, typically resulting in the formation of oligomers rather than high-molecular-weight polymers. Consequently, no efficient ADMET polymerizations of diallyl succinate (**DAS**), diallyl adipate (**DAA**), diallyl terephthalate (**DAT**), or diallyl carbonate (**DAC**) monomers have been reported to date. In this study, the ADMET activity of the catalysts depicted in Scheme 2 was evaluated using **DAS** as a model monomer (Scheme 3, top).

**Scheme 2:**
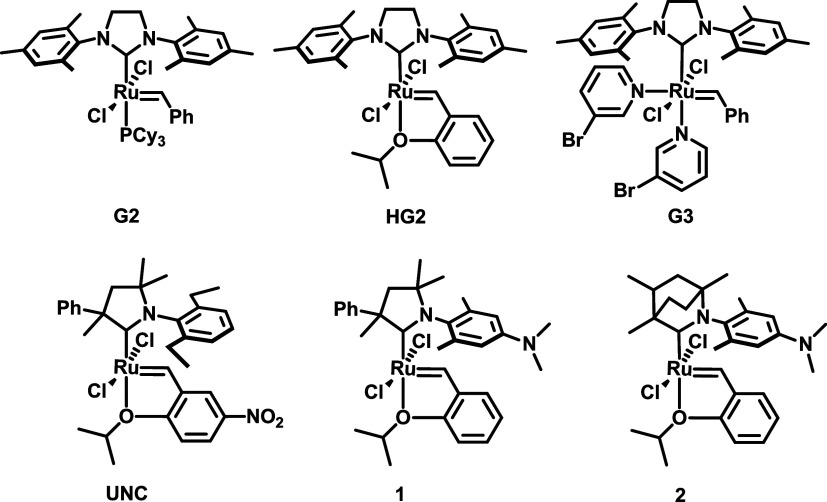
Catalysts Tested in ADMET Reactions of Diallyl Esters Depicted in Scheme 3

**Scheme 3:**
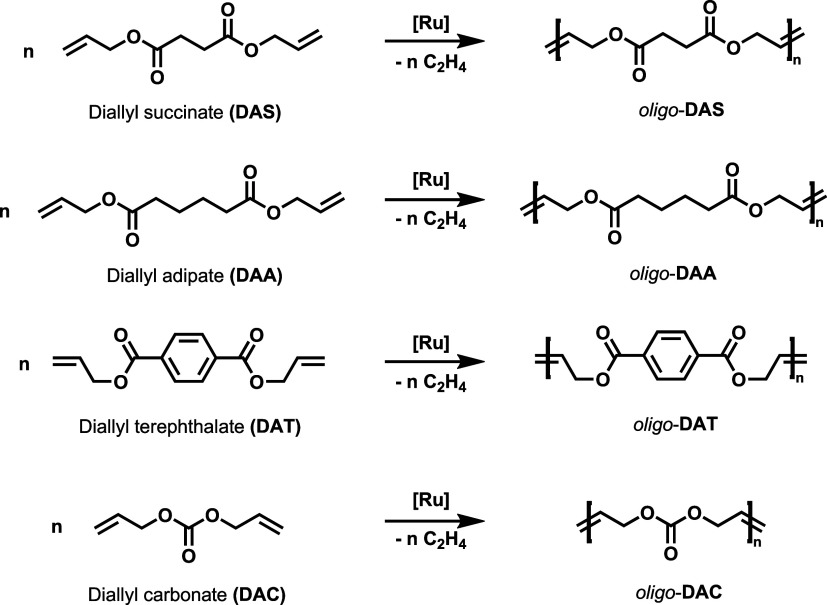
Acyclic Diene Metathesis (ADMET) Oligomerization of Diallyl Succinate (**DAS**), Diallyl Adipate (**DAA**), Diallyl Terephthalate (**DAT**), and Diallyl Carbonate (**DAC**)­^a^

a Conditions: 0.1 mol % catalyst, room-temperature (RT), 3 h, toluene ([substrate] = 2.5 M).

The reactions were carried out using 0.1 mol % catalyst loading at room temperature for 3 h (see SI). It was found that catalysts **2** and **G2** were inactive in the ADMET reaction, meanwhile **1** and **G3** showed some monomer conversion (46% and 17%, respectively). The limited catalytic activity of **G3** can be attributed to the intrinsic instability of its methylidene intermediate, formed during the reaction of **G3** with ethylene or α-olefins. Fogg and co-workers demonstrated that pyridine-containing methylidene complexes are stable only at very low temperatures (−78 °C), but they decompose at measurable rates even at −10 °C and are prone to degradation in the solid state as well.^35,36^ Surprisingly, both **HG2** and **UNC** exhibited reasonable catalytic activity, likely due to their rapid initiation capability, achieving monomer conversions of 72% and 82%, respectively. Despite the relatively high conversions and broad molecular mass dispersities (*Đ*_M_ = 2.25 and 2.16), the resulting oligomers displayed low molecular weights (*M*_w_ = 1.0 and 1.6 kDa).

According to these preliminary results, the most active **UNC** catalyst was used for all further ADMET oligomerization reactions (Scheme 2).

The ADMET oligomerizations of **DAS**, **DAA**, **DAT**, and **DAC** were performed under analogous conditions, employing 0.1 mol % **UNC** catalyst loading in toluene at room temperature (Table 1). Interestingly, a high yield but low *M*_w_ was observed for **DAT** (Table 1, entry 3), which could be explained by the poor solubility and thus the immediate precipitation of the oligomers even at diluted conditions (0.05 M). In the other cases (Table 1, entries 1, 2, and 4) no precipitation was observed, only the formation of a viscous reaction mixture. Notably, the diallyl esters and diallyl carbonate exhibited no ADMET activity at low temperature (0 °C).

**Table 1:** ADMET Oligomerization of Diallyl Esters Using **UNC** Catalyst^a^

entry	polymer	yield [%]	*M*_w_ [kDa]	*Đ*_M_	DP^b^	DP^c^
1	*oligo*-**DAS**	82	1.6	2.16	4.2	6.4
2	*oligo*-**DAA**	65	2.2	1.94	5.6	6.7
3	*oligo*-**DAT**	98	1.0	1.92	2.2	3.0
4	*oligo*-**DAC**	75	1.8	2.01	7.4	8.3
5^d^	*oligo*-**DAT**	93	1.0	1.25	4.0	4.0
6^d^	*oligo*-**DAA**	94	8.3	6.38	7.0	6.0

a Reaction conditions are given in Scheme 2.

b Degree of polymerization (DP) determined by APC (DP = *M*_n_/monomer molecular weight).

c DP is determined by ^1^H NMR.

d Data from ref 37 (multiple catalyst addition).

### 2.2 Cyclic–Acyclic Monomers Metathesis Polymerization (CAMMP) of Cyclopentene (**CP**) with Diallyl Esters

**CP** was copolymerized with allyl esters at a 10:1 ratio using **UNC** catalyst in toluene solution at room temperature (Scheme 4 and Table 2, entries 1-4). The copolymers were precipitated by methanol, giving white solids in reasonable yield (75–97%). The observed conversions are slightly higher than those obtained for **CP** homopolymers prepared via equilibrium polymerization under similar conditions (approximately 50% under the reported conditions in the absence of diallyl esters),^38^ indicating that **CP** is less susceptible to equilibrium limitations during CAMMP^39^ copolymerization. While lower temperatures are generally favorable for ROMP of **CP** owing to its equilibrium-driven nature, the absence of detectable ADMET activity at 0 °C necessitated carrying out the CAMMP reaction at room temperature, where efficient conversion could be achieved.

**Scheme 4:**
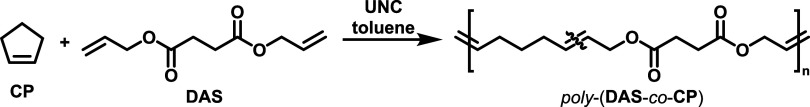
CAMMP of Diallyl Esters (e.g., **DAS**) and **CP** ^a^

a Conditions: 0.1 mol % catalyst, RT, 3 h, toluene (20 vol % **CP**).

**Table 2:** CAMMP of Diallyl Esters and ROIMP of *Oligo*-Diallyl Esters with **CP** ^a^

entry	polymer	CP/DAE ratio^d^	yield [%]^e^	*M*_w_	*Đ*_M_	*T*_d_ [°C]	**CP**/ester ratio^f^,^h^
1^b^	CAMMP-*poly*-(**DAS**-*co*-**CP**)-**10**	10	75	4.1	2.56	ND	6 (94%)
2^b^	CAMMP-*poly*-(**DAA**-*co*-**CP**)-**10**	10	81	3.1	2.04	ND	3 (89%)
3^b^	CAMMP-*poly*-(**DAT**-*co*-**CP**)-**10**	10	97^g^	4.2	2.13	ND	4 (95%)
4^b^	CAMMP-*poly*-(**DAC**-*co*-**CP**)-**10**	10	84	3.2	2.09	ND	5 (94%)
5^c^	ROIMP-*poly*-**(DAS**-*co*-**CP)-10**	10	67	9.2	1.94	206	6 (72%)
6^c^	ROIMP-*poly*-**(DAA**-*co*-**CP)-10**	10	61	11	1.88	225	5 (85%)
7^b^	ROIMP-*poly*-**(DAT**-*co*-**CP)-10**	10	81	8.8	4.22	ND	4 (65%)
8^c^	ROIMP-*poly*-**(DAC**-*co*-**CP)-10**	10	63	14	2.01	200	5 (61%)
9^c^	ROIMP-*poly*-**(DAS**-*co*-**CP)-100**	100	93	27	1.71	312	48 (74%)
10^c^	ROIMP-*poly*-**(DAA**-*co*-**CP)-100**	100	87	32	1.37	325	36 (96%)
11^c^	ROIMP-*poly*-**(DAT**-*co*-**CP)-100**	100	95^g^	11	3.12	ND	73 (83%)
12^c^	ROIMP-*poly*-**(DAC**-*co*-**CP)-100**	100	89	26	1.42	291	39 (82%)

a Toluene (20 vol % CP), UNC = 0.1 mol %, *t*_r_ = 3 h.

b 25 °C.

c 0 °C.

d **CP**/diallyl estermonomer ratio.

e Isolated yield—The polymer yield was calculated based on the maximum theoretical equilibrium polypentenamer yield of 55% at 25 °C and 82% at 0 °C.^38^

f Determined by ^1^H NMR.

g Polymer precipitation was observed.

h Percentage of singular **DAS**, **DAA**, **DAT**, and **DAC** dyads in parentheses. ND: no data.

The random monomer distribution has been indicated by homonuclear (^1^H–^1^H TOCSY, ^1^H–^1^H COSY) and heteronuclear (^1^H–^13^C HSQC and ^1^H–^13^C HMBC) measurements (Figures S2–S5). Interestingly, the formed oligomers contained mainly singular diallyl ester units (>89%, Table 2, entries 1–4), and only a minor amount of ester block was detected. The molecular weights (*M*_w_) and dispersity indices (*Đ*_M_) were investigated by advanced polymer chromatography (APC) indicating some molecular weight improvement compared with the ADMET homopolymers of allyl esters. The *Đ*_M_ in each case remained high (*Đ*_M_ > 2; Table 2, entries 1–4). However, the *M*_w_ values of the copolypentamers were still far below those values obtained in the absence of diallyl esters under similar conditions (*M*_w_ = 3.2–4.2 kDa at RT vs 31.8 kDa at 30 °C).^38^

### 2.3 Synthesis of Cleavable Unit Containing Polypentenamers (PPe) via Ring-Opening Insertion Metathesis Polymerization (ROIMP)

The ROIMP reactions of *oligo*-(**DAS**, **DAA**, **DAT**, and **DAC**) with **CP** were investigated in toluene solution. In contrast to ADMET, which proceeds via a step-growth condensation mechanism, the ring-opening metathesis polymerization (ROMP) of **CP** was conducted at 0 °C. Due to the moderate ring strain of cyclopentene (5.4 kcal mol^–1^), its ROMP is both exothermic and equilibrium-controlled. Accordingly, lowering the reaction temperature shifts the equilibrium toward polymer formation, whereas elevated temperatures promote depolymerization.

In the first series of experiments, the ratio of diallyl ester units and applied **CP** monomers was set to 1:10. After three hours reaction time, the polymers were precipitated by methanol as white, dense, waxy solids. As expected, insertion of the **CP** ring into the olefinic double bonds of the diallyl ester oligomers was observed (Scheme 5). In contrast to the CAMMP copolymerization of **CP** with diallyl esters, the ROIMP copolymerization of **CP** with *oligo*-(**DAS**), *oligo*-(**DAA**), *oligo*-(**DAT**), and *oligo*-(**DAC**) yielded copolymers with significantly higher molecular weights (*M*_w_) (*M*_w_ = 8.8–14 kDa vs *M*_w_ = 3.2-4.2 kDa; ROIMP-*poly*-(**DAS**, **DAA**, **DAT**, and **DAC**-*co*-**CP**)-**10**; Table 2, entries 5-8). Based on ^1^H NMR investigations, it was found that the formed polymers contained relatively small amounts of ester–ester/carbonate–carbonate dyads (Figure 1, H^*e*^ signal; Figures S10-S13). This indicates that during the ROIMP reaction, no block polymers containing polyester and **PPe** dyads formed, but rather **PPe** containing randomly distributed singular ester/carbonate units. Interestingly, it was also found that not only the internal but also the terminal double bonds of the oligoesters and oligocarbonate can participate in the ROIMP reaction, which could be clearly seen by the upshift of the multiplet proton signals from 5.2 to 4.9 ppm (Figure 1, H^*b*^ signal; also see details in the SI, Section 5.3). The assignation of H^*i*^ and H^*h*^ protons was confirmed by 2D NMR correlation spectroscopy (COSY; see SI, Section 3).

**Figure 1:**
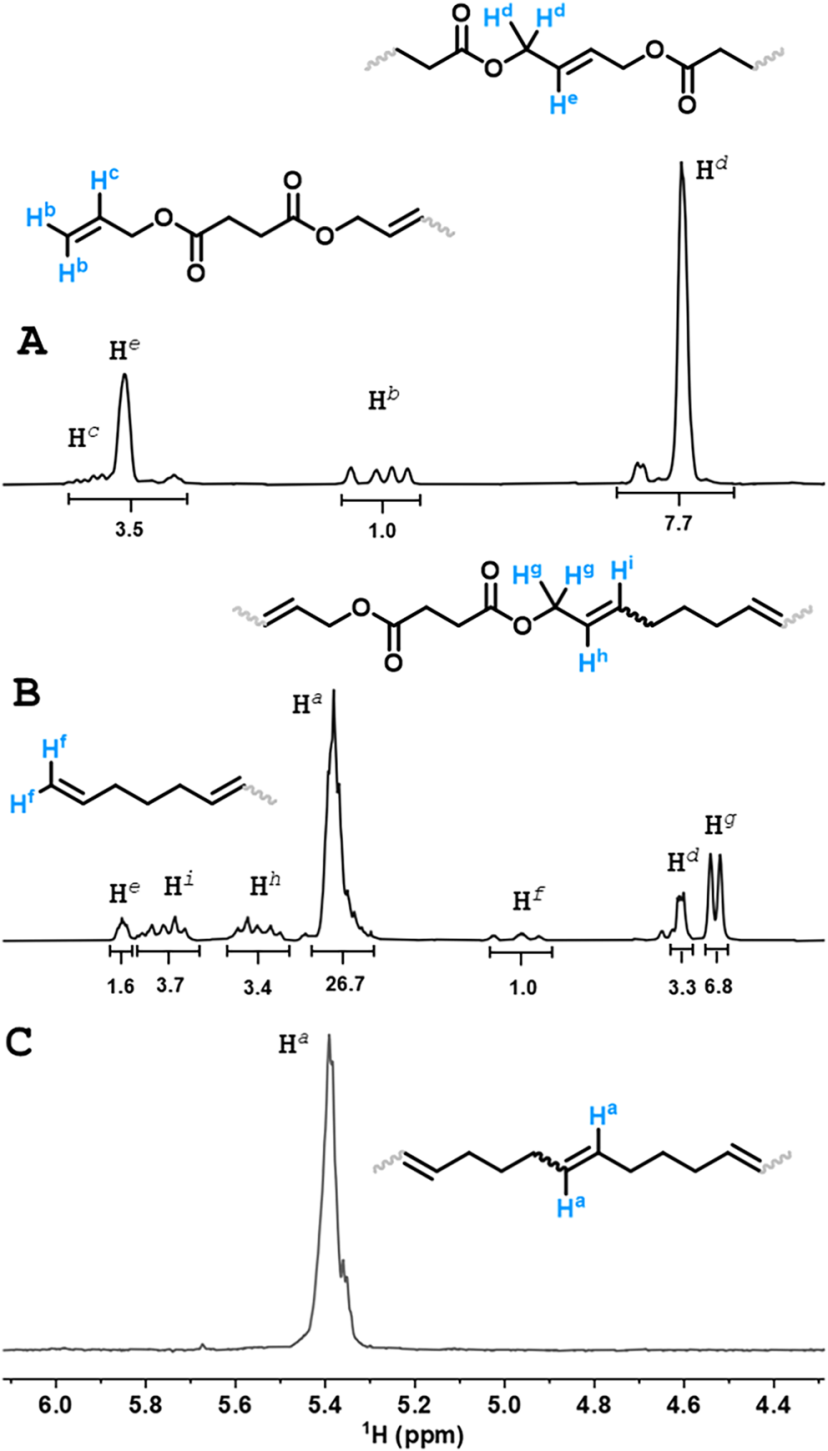
Representative example for ^1^H NMR analysis of ROIMP polymerization. Stacked ^1^H NMR spectra of *oligo*-**DAS** oligomer (A), ROIMP-*poly*-**(DAS**-*co*-**CP)-10** (B), and polypentenamer (**PPe**) (C) (CDCl_3_).

**Scheme 5:**
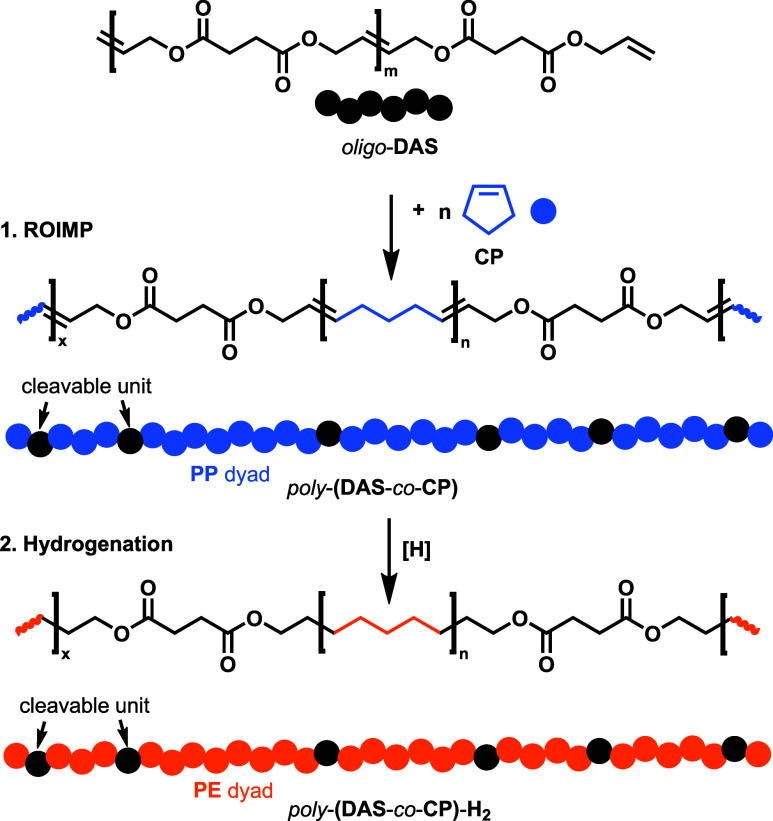
Synthesis of Saturated and Polyunsaturated Hydrocarbon Polymers via Ring-Opening Insertion Metathesis Polymerization (ROIMP) of **CP** with o*ligo*-**DAS**

Increasing the ratio of **CP** by 1 order of magnitude, the formation of **PPe** copolymers with even higher molecular weight was observed (*M*_w_ = 26-32 kDa; ROIMP-*poly*-**(DAS**, **DAA**, **DAT**, and **DAC**-*co*-**CP)-100**; Table 2). These tests clearly indicated that increasing the applied **CP** ratio results in a significant increase of the copolymer molecular weight. Furthermore, based on ^1^H NMR analysis, for example in ROIMP-*poly*-(**DAA**-*co*-**CP**)-**100** polymers, **DAA** oligomer dyads at 5.85 ppm could not be detected at all; only singular **DAA** units at 5.60-5.80 ppm were detected (Figure S12). It should be noted that among the tested oligomers, ROIMP with *oligo*-**DAA** resulted in the highest molecular weight polymer (*M*_w_ = 32 kDa), giving reasonable dispersity (*Đ*_M_ = 1.37) with a high singular **DAA** ratio (96%) in 87% yield.

TGA analysis indicated that higher **CP** monomer content results in higher thermostability of the ROIMP copolymers. For example, polymer ROIMP-*poly*-(**DAA**-*co*-**CP**)-**10** has shown less thermostability compared to ROIMP-*poly*-(**DAA**-*co*-**CP**)-**100**. Its degradation starts above 225 °C, while the latter starts to decompose at considerably higher temperatures (325 °C, Table 2, entries 5-12). Both samples show a two-step decomposition pathway, whereas in both cases the first step (approximately between 250 and 400 °C) is exothermic, while the second, larger one is endothermic. Despite the inert (pyrolytic) conditions used in the measurements, both sample’s decomposition ends at the same temperature (492 °C) and is nearly quantitative (residue at the end of the measurement for sample ROIMP-*poly*-(**DAA**-*co*-**CP**)-**10** is 1%, while for the ROIMP-*poly*-(**DAA**-*co*-**CP**)-**100** is 2.5%; see SI, Section 8).

### 2.4 Hydrolytic Cleavage of PPe Copolymer Elastomers

Hydrolysis studies of ROIMP copolymers (Scheme 6 and Table S1) were performed under alkaline conditions. The copolymers were dissolved in THF followed by addition of an aqueous solution of sodium hydroxide (2 M). The mixture was refluxed overnight. The solution was then neutralized by addition of hydrochloric acid resulting in telechelic, hydroxyl group end-functionalized **PPe** chains in high yield (61-99%). APC analysis of the formed mixture revealed a significant drop of the *M*_n_ while the *Đ*_M_ increased dramatically compared to the starting polymers. In a typical example based on the ^1^H NMR and ^1^H-^13^C HMBC measurements, the ROIMP-*poly*-(**DAS**-*co*-**CP**)-**100** polymer could be completely hydrolyzed to telechelic **PPe** diols (Figures 2 and S16; Table S1, entry 1), while the *M*_n_ of the starting polymer dropped from 15.6 to 2.8 kDa and the *Đ*_M_ increased from 1.71 to 4.25. The HMBC analysis has shown that there is no correlation between the α-position protons and carbonyl carbon, indicating that at the end of the reaction, the products do not contain an ester group. Considering that the polymerization degree of the starting *oligo*-**DAS** oligomer used in the ROIMP reaction is around 4-6 (Table 2), after the hydrolysis of the ROIMP-*poly*-(**DAS**-*co*-**CP**)-**100** polymer the *M*_n_ value of the telechelic **PPe** fragments should be approximately one-fifth of the starting polymer *M*_n_ value, which is in good agreement with the observed APC *M*_n_ data (Figures 2 and S15). The broad dispersity of the telechelic polypentenamer aligns with the assumption that the ester/carbonate units are randomly distributed in the ROIMP polymers.

**Figure 2:**
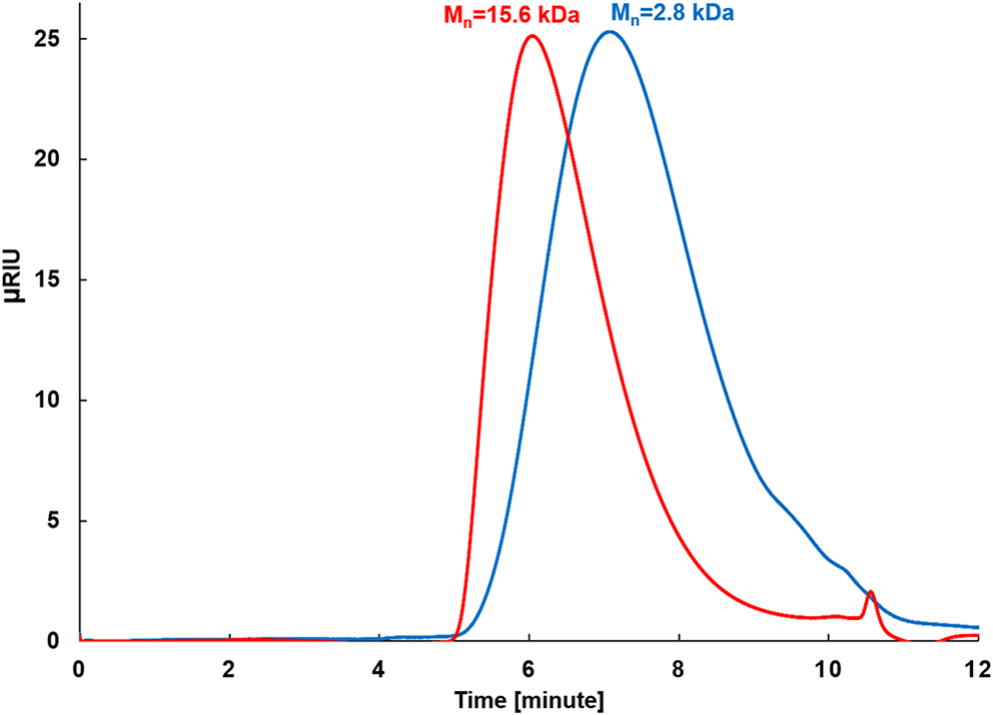
APC investigation of ROIMP-*poly*-(**DAS**-*co*-**CP**)-**100** polymer: before (red) and after hydrolysis (blue).

**Scheme 6:**
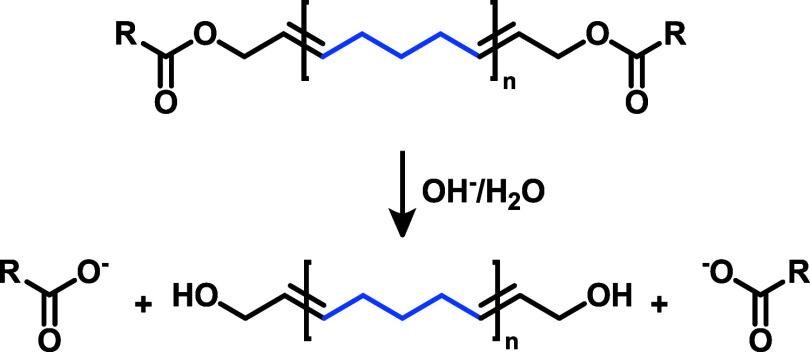
Hydrolysis of Ester Group-Containing Polypentenamer (**PPe**) Leading to Telechelic Hydroxyl End-Group-Functionalized **PPe** Oligomers^a^

a 50 mg of polymer in THF (25 mg/mL), 2 mL of 2 M NaOH, refluxed, *t*_r_ = 16 h.

### 2.5 Hydrogenation of Ester/Carbonate Unit Containing PPe Synthesized by ROIMP

The synthesized ROIMP **PPe** copolymers were hydrogenated using Wilkinson’s catalyst in THF solution at 50 °C and 20 bar hydrogen pressure (Scheme 5). During the reaction, the hydrogenated polymers gradually precipitated from the solution. The obtained polymers contained saturated, long hydrocarbon chains, which made the 1:100 copolymers insoluble, while the 1:10 copolymers were poorly soluble in chloroform and dichloromethane. ^1^H NMR analysis of the hydrogenated 1:10 copolymer confirmed successful hydrogenation of the polymer backbone; however, residual unsaturated moieties were still detected, as evidenced by characteristic signals in both the ^1^H NMR and IR spectra. This observation is likely attributed to the precipitation of partially hydrogenated polymer chains during the reaction, which may have limited their full exposure to the hydrogenation catalyst (Figure S20B). Furthermore, the ^1^H-^13^C HMBC measurement showed that the methylene group next to the oxygen still correlates with the carbonyl group of the polymer chain after hydrogenation, which confirms the presence of ester groups in the polymer chain (Figure S20C).

To demonstrate that the ester and carbonate groups remain intact during hydrogenation, we performed the reduction of the ADMET polyester and polycarbonate oligomers. ^1^H NMR measurements did not show a significant change in the degree of polymerization of the oligomers compared to the unsaturated starting oligomers (Table S2), which indicates that the ester or carbonate functional groups in the polymer chain are less affected during the hydrogenation. The effect of the hydrogenation of sample ROIMP-*poly*-(**DAA**-*co*-**CP**)-**100** is clearly visible on both comparative graphs (mass loss/TGA/and heat flow/DSC/). As expected, the most obvious difference is the further increase in the thermal stability of the hydrogenated sample. Its thermal decomposition starts at 400 °C, more than 50 °C higher, compared to the corresponding non-hydrogenated sample (325 °C). The decomposition of the sample ends at 500 °C, and in this case too, it is also almost quantitative (residue at the end of the measurement for sample ROIMP-*poly*-(**DAA**-*co*-**CP**)-**100-H**_**2**_ is 1.5%) (Figure S24 and Table S3). The glass transition temperatures (*T*_g_) of the synthesized polymers were outside the examined range. According to the literature, **HDPE** typically exhibits a *T*_g_ between approximately −100 and −130 °C,^40^ whereas for polypentenamers, this value is around −44 °C.^41^

### 2.6 Polymerization on Multigram Scale and Investigation of Polymer Physical Properties

The synthesis of ROIMP copolymers was successfully performed on a multigram scale using **UNC** as the ADMET catalyst and **HG2** as the ROIMP catalyst. The ADMET reaction afforded *oligo*-**DAS** with molecular weight and yield comparable to those obtained in small-scale experiments (*M*_w_ = 5 kDa; 56%). Subsequent ROIMP of *oligo*-**DAS** with **CP** at a **CP**-to-succinate ratio of 100 resulted in a substantial increase in molecular weight (*M*_w_ = 22 kDa; ROIMP-*poly*-(**DAS**-*co*-**CP**)-**100**) with a good yield (64%) and maintained a reasonable dispersity (*Đ*_M_ = 1.40). These values are slightly lower than those observed in the small-scale synthesis (*M*_w_ = 27 kDa; *Đ*_M_ = 1.71; Table 2, entry 9).

^1^H NMR analysis indicated that the ratio of cyclopentene to ester units in the resulting ROIMP copolymer was approximately 80 per succinate unit (40 per ester dyad), consistent with the theoretically calculated **CP** incorporation per succinate assuming ROMP of **CP** reached equilibrium (82% at 20 vol % **CP** in toluene at 0 °C).^38^ Repeating the ROIMP reaction with an additional 100-fold portion of **CP** and **HG2** catalyst led to a modest increase in molecular weight (*M*_w_ = 27 kDa; ROIMP-*poly*-(**DAS**-*co*-**CP**)-**200**), while dispersity remained essentially unchanged (*Đ*_M_ = 1.40). The isolated copolymers were obtained in reasonable yield (56%), and as expected, the ^1^H NMR confirmed that the **CP**-to-succinate ratio approximately doubled to 180 (90 per ester dyad).

The ROIMP copolymer was subsequently hydrogenated in a 700 mL stainless steel autoclave using Wilkinson’s catalyst in THF (for details, see SI, Section 9), yielding the saturated polymer in high yield (78%). It is presumed, based on previous ^1^H NMR analysis of ROIMP-*poly*-(**DAS**-*co*-**CP**)-**10-H2** (Figure S20B), that approximately 95% of the olefinic double bonds are hydrogenated.

Infrared spectra recorded in the ATR mode are shown in Figure 3a. A commercial grade **HDPE** (Melt Index ≥ 10.5, *M*_w_ = 72.4 kDa) was used as a reference. Based on the attenuated total reflectance-Fourier transform infrared spectroscopic (ATR-FTIR) analysis, it can be concluded that the non-hydrogenated elastomer exhibits the characteristic vibrations associated with carbon–carbon double bonds, as expected: the absorptions observed between 3050 and 3000 cm^–1^ correspond to the symmetric stretch of = CH– structural units, while the intense peak at 965 cm^–1^ is likewise attributed to the =CH– bending vibration of the olefinic unit.^42^ The figure clearly shows that the intensity of these bands decreases markedly upon hydrogenation; however, they do not completely vanish, indicating the presence of residual unsaturation consistent with earlier ^1^H NMR analysis.

**Figure 3:**
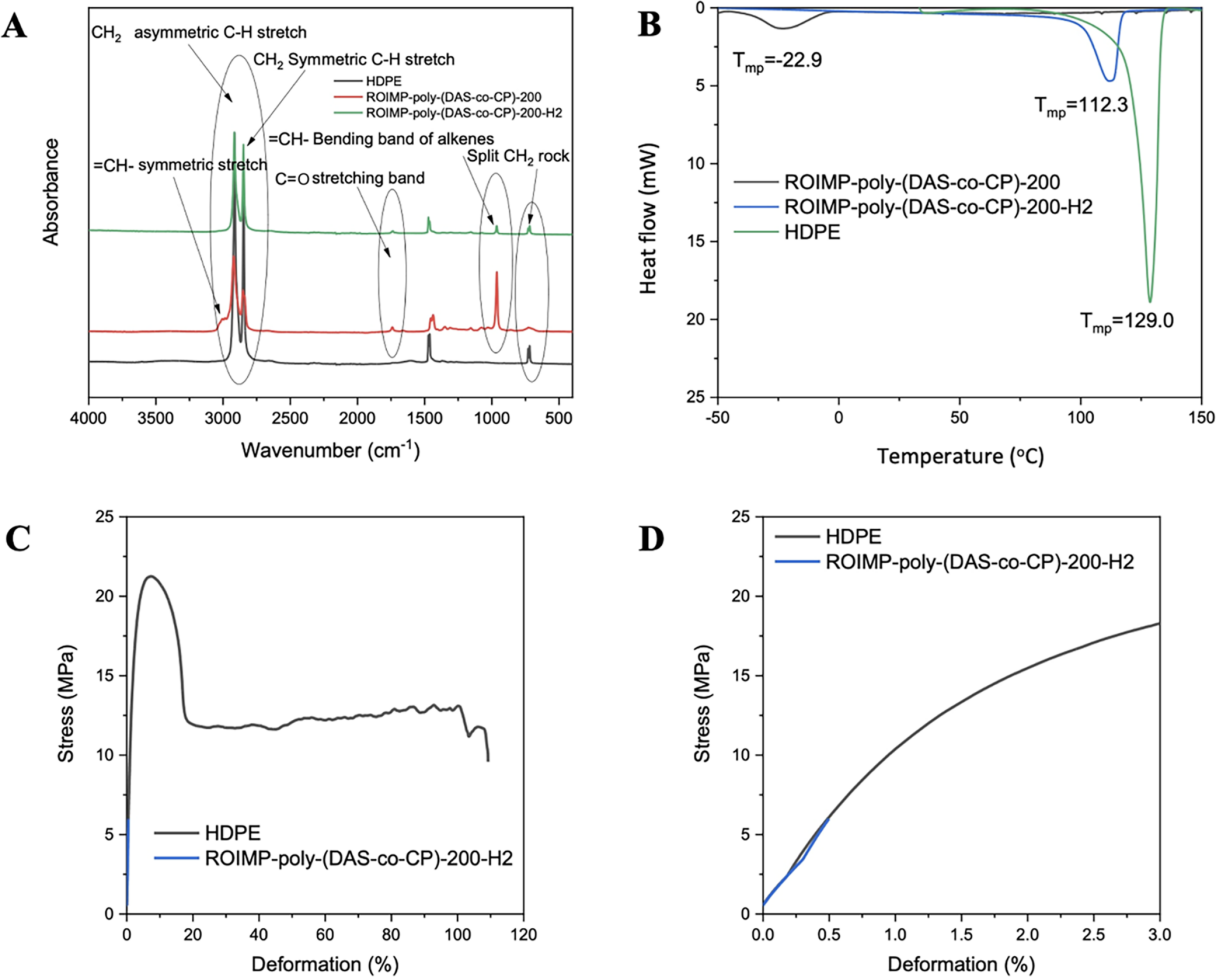
(a) FTIR-ATR spectra of ROIMP-*poly*-(**DAS**-*co*-**CP**)-**200**, ROIMP-*poly*-(**DAS**-*co*-**CP**)-**200-H**_**2**,_ and the reference **HDPE** samples, (b) differential scanning calorimetry (DSC) traces of different polypentenamer samples, (c) Stress–strain curves of the synthesized materials and the reference **HDPE** sample, and (d) magnification of the initial part of the stress–strain curve.

The peaks observed at 729 and 719 cm^–1^ are also characteristic of polyethylene materials, more specifically of linear polyethylene.^43^ These bands correspond to the rocking vibrations of CH_2_ units located in the crystalline phase, which are barely detectable in the non-hydrogenated elastomer due to its low melting temperature. The C=O stretching band detected between 1750 and 1725 cm^–1^ appears in all samples except the **HDPE** reference, indicating the presence of carboxyl groups in the synthesized **ROIMP**-*poly*-**(DAS**-*co*-**CP)-200** and **ROIMP**-*poly*-**(DAS**-*co*-**CP)-200-H**_**2**_ polymers (Figure 3a).

DSC measurements clearly show that while the non-hydrogenated elastomer exhibits a low melting temperature, the hydrogenated samples present relatively high melting points (Figures 3b and S29). The even higher melting temperature of **HDPE** can be explained by its greater chain regularity. According to the Sanchez–Eby inclusion model, polymers containing randomly distributed ester units along the chain exhibit a linear decrease in melting temperature (112.3 °C).^44^

The mechanical properties of the synthesized samples, primarily their deformability and tensile strength, fall short of the performance of the **HDPE** reference (Figure 3c). This can be attributed to the high-temperature compression molding required for specimen preparation, and the materials did not contain any antioxidants. Additionally, the unsaturated segments present in the materials make them susceptible to oxidation, leading to failure at relatively low deformations. However, for the higher-molecular-weight sample, it can be stated that its elastic modulus approaches that of reference **HDPE** (Figure 3d).

## 3 Conclusions

Ester- and carbonate-containing polyolefins were synthesized via the ROIMP of unsaturated polyester oligomers with cyclopentene (**CP**). Initially, the ADMET polymerization of diallyl esters—including diallyl succinate, adipate, terephthalate, and carbonate—was developed using ruthenium-based metathesis catalysts. Although high monomer conversions were achieved in all cases, ADMET polymerization using **UNC**, one of the most effective catalysts, yielded oligomers of relatively low molecular weight (0.5–2.2 kDa). Subsequent ROIMP copolymerization of the isolated oligomers with **CP** produced polypentenamer chains incorporating randomly distributed, singular ester or carbonate functionalities. Hydrolysis of these copolymers afforded telechelic, hydroxyl-terminated polypentenamer mixtures with low *M*_n_ and increased dispersity (*Đ*_M_). Hydrogenation of the ROIMP copolymers yielded saturated hydrocarbon polymers containing ester units. Upscaling of the ROIMP copolymerization methodology was demonstrated on the copolymerization of **CP** with *oligo*-**DAS**. Although thermal, deformability, and mechanical strength analyses revealed certain similarities to **HDPE**, the hydrogenation of the polyunsaturated polymers still requires improvement, and optimization efforts are currently underway.

## Supplementary Material



## Notes

Detailed experimental conditions, materials, and methods as well as supplemental NMR, APC and thermoanalytical data for all polymers (PDF)
