# Supplementary material for: Synthesis of Cleavable Polyolefins via Ring-Opening Insertion Metathesis Polymerization (ROIMP) of Cyclopentene with Unsaturated Polyester Oligomers
Source: Macromolecules. 2025 Dec 22;59(1):515–23. doi: 10.1021/acs.macromol.5c03036 (PMC12805637; doi:10.1021/acs.macromol.5c03036)
Supplement: Supplementary file 1 [file ma5c03036_si_001.pdf]

Supporting Information

Synthesis of Cleavable Polyolefins via Ring-Opening Insertion Metathesis Polymerization (ROIMP) of Cyclopentene with Unsaturated Polyester Oligomers

*Vajk Farkas, Ádám Erdélyi, Kristóf Varga, Dang Vu Hai, Momoko Ishii, Márton Nagyházi, Gábor Turczel, Pooja Dubey, László Trif, Ole Osterthun, Paul T. Anastas, Jürgen Klankermayer, János Moczó and Róbert Tuba**

Table of Contents

1. General Information 1

2. Monomer synthesis 2

3. CAMMP copolymerization 3

3.1. Representative example of CAMMP copolymerization 3

3.2. NMR analysis of CAMMP copolymerizations 5

4. ADMET homopolymerization 9

4.1. Representative example of ADMET homopolymerization 9

4.2. NMR of ADMET homopolymers 9

5. ROIMP copolymerization 11

5.1. Representative example of ROIMP copolymerization in 10:1 ratio 11

5.2. Representative example of ROIMP copolymerization in 100:1 ratio 12

5.3. NMR analysis of ROIMP copolymerization 12

6. Hydrolysis 18

7. Hydrogenation of oligomers and ROIMP-copolymers 19

7.1. Representative example of hydrogenation of ROIMP-copolymers 19

7.2. NMR analysis of hydrogenated oligomers 21

7.3. NMR analysis of hydrogenated ROIMP-copolymers 23

8. Representative example for comparison of TGA diagrams before and after hydrogenation. 26

9. Scaled-up synthesis of ROIMP-*poly*-(DAS-*co*-CP)-100 and ROIMP-*poly*-(DAS-*co*-CP)-200 27

9.1. Scaled-up ADMET homopolymerization 27

9.2. Scaled-up ROIMP copolymerization of poly-DAS in 100:1 ratio 27

9.3. Scaled-up ROIMP copolymerization of ROIMP-*poly*-(DAS-co-CP)-100 28

9.4. Scaled-up hydrogenation of ROIMP-*poly*-(DAS-*co*-CP)-200 30

10. Physical properties 31

11. References...34

1. General Information

All metathesis reactions were conducted under nitrogen atmosphere using Schlenk-technique or under argon using a glovebox. $CDCl_3$, reagents and solvents (Aldrich) were used as received.

Solution-state NMR spectra were acquired using Varian NMR System spectrometers operating at 300, 400 and 600 MHz respectively. Notation for the ^{1}H NMR spectral splitting patterns includes singlet (s), doublet (d), triplet (t), broad (br) and multiplet/overlapping peaks (m). Chemical shifts (δ values) are given in ppm, coupling constants (J) are expressed in Hertz. ^{1}H and ^{13}C NMR assignment of the diester-cyclpentene copolymers were obtained using the combination of two-dimensional homonuclear (^{1}H-^{1}H TOCSY, ^{1}H-^{1}H COSY) and heteronuclear (^{1}H-^{13}C HSQC and ^{1}H-^{13}C HMBC) measurements.

APC measurements were carried out using Acquity Advanced Polymer Chromatography System and Waters 2414 Refractive Index detector at 45 ˚C and columns at 30˚. The sample manager was used at 25 ˚C. Column manager container three columns in series, (4.6×150 mm): ACQUITY APCTM XT 200 Å 1.7 μm, ACQUITY APCTM XT 125 Å 2.5 μm, ACQUITY APCTM XT 45 Å 1.7 μm. The third order calibration curve was used, and polystyrenes standards (66000 Da, 21500 Da, 4920 Da, 2280 Da) were used for calibration. HPLC grade THF was used as eluent (VWR). The flow rate was 0.5 mL/min. For the calculations of the molecular weights, Empower Chromatography Data System was used. The concentration of the sample was 1 mg/mL, and the volumes of injections were 50 μL

Thermal measurements were performed on a Setaram LabsysEvo (Lyon, France) TG-DSC system, in flowing high purity nitrogen (99.999%, flow rate 90 mL/min) atmosphere. Samples were weighed into 100 μL alumina crucibles (the reference cell was empty) and where heated from 25 ˚C to 600 ˚C with a heating rate of 10 ˚C/min. The obtained data was blank corrected and further processed with the thermoanalyzer's processing software (Calisto Processing v2.15, AKTS, Switzerland). The thermal analyzer (both the temperature scale and calorimetric sensitivity) was

calibrated by a multipoint calibration method, in which seven different certified reference materials were used to cover the thermal analyzer's entire operating temperature range.

Flash column chromatography was performed on a CombiFlash Rf 150 apparatus using gradient elution in normal (silica hexane−ethyl acetate) phase mode. Sample loadings were performed in the case of silica flash chromatography by coating the sample onto a silica cartridge.

Yield was calculated in the case of ADMET, as the following:

$$yield = \frac{m(isolated\ polymer)}{m(diallyl\ ester) - n(diallyl\ ester) * M(ethylene)} * 100\%$$

Yield was calculated in the case of CAMMP, as the following:

$$yield = \frac{m(isolated\ polymer)}{m(CP) + m(diallyl\ ester) - n(diallyl\ ester) * M(ethylene)} / reqc * 100\%$$

Yield was calculated in the case of ROIMP reaction as the following:

$$yield = \frac{m(isolated\ polymer)}{m(CP) + m(oligomer)} / reqc * 100\%$$

where m(isolated polymer) is the mass of the isolated polymer; m(diallyl ester) is the mass of the diallyl ester; n(diallyl ester) is the molar amount of diallyl ester; M(ethylene) is the molecular weight of ethylene; m(CP) is the mass of the cyclopentene; m(oligomer) is the mass of the previously prepared oligomers derived from diallyl esters; *reqc* is a constant to describe the relative equilibrium composition of the ring-opening polymerization reaction of **CP** at different temperatures, for example at 0°C 82% of the cyclopentadiene exist as polycyclopentadiene in an equilibrium mixture and as such req=0,82 at 0°C.

2. Monomer synthesis

The synthesis of **DAA** and **DAS** followed the same procedure as reported by Yang for the formation of **DAT**.[1] The NMR spectra of **DAA**[2] and **DAS**[3] were identical to those in the literature.

3. CAMMP copolymerization

3.1. Representative example of CAMMP copolymerization

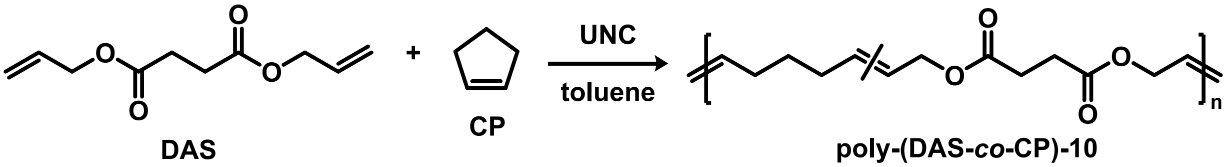

Diallyl succinate (100 mg, 0.5 mmol, 1 eq.) and cyclopentene (462 µL, 5.0 mmol, 10 eq.) were placed in a vial and were dissolved in toluene (1.5 mL) using a stirring bar and toluene solution of **UNC** (3.8 mg, 5.5 µmol, 0.1 mol% in 380 µL toluene) was added. The mixture was stirred at RT for 3 hours. After that the formed precipitation was dissolved in THF, followed by the addition of ethyl vinyl ether (0.1 mL). The mixture was stirred for 10 minutes, and MeOH was added to precipitate the polymer (183 mg, 41%).

To confirm the random copolymerization between the diesters and cyclopentene, two-dimensional NMR experiments were conducted in $CDCl_3$. This involved a combination of homonuclear (^{1}H-^{1}H TOCSY, ^{1}H-^{1}H COSY) and heteronuclear (^{1}H-^{13}C HSQC and ^{1}H-^{13}C HMBC) measurements to obtain assignments for the different linked moieties.

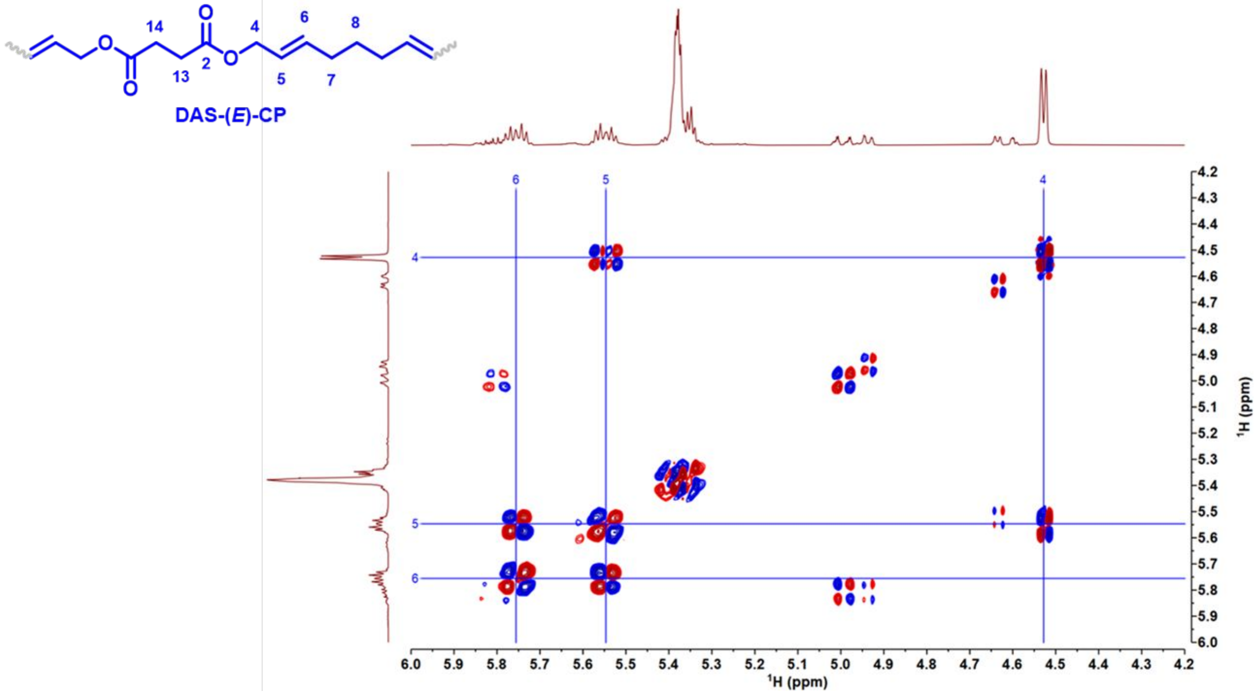

Figure S1. Expansion of the ^{1}H-^{1}H COSY spectra of copolymer *poly*-(**DAS**-*co*-**CP**)-**10**, illustrating the ^{1}H-^{1}H connectivities in the detected **DAS-(*E*)-CP** moiety.

3.2. NMR analysis of CAMMP copolymerizations

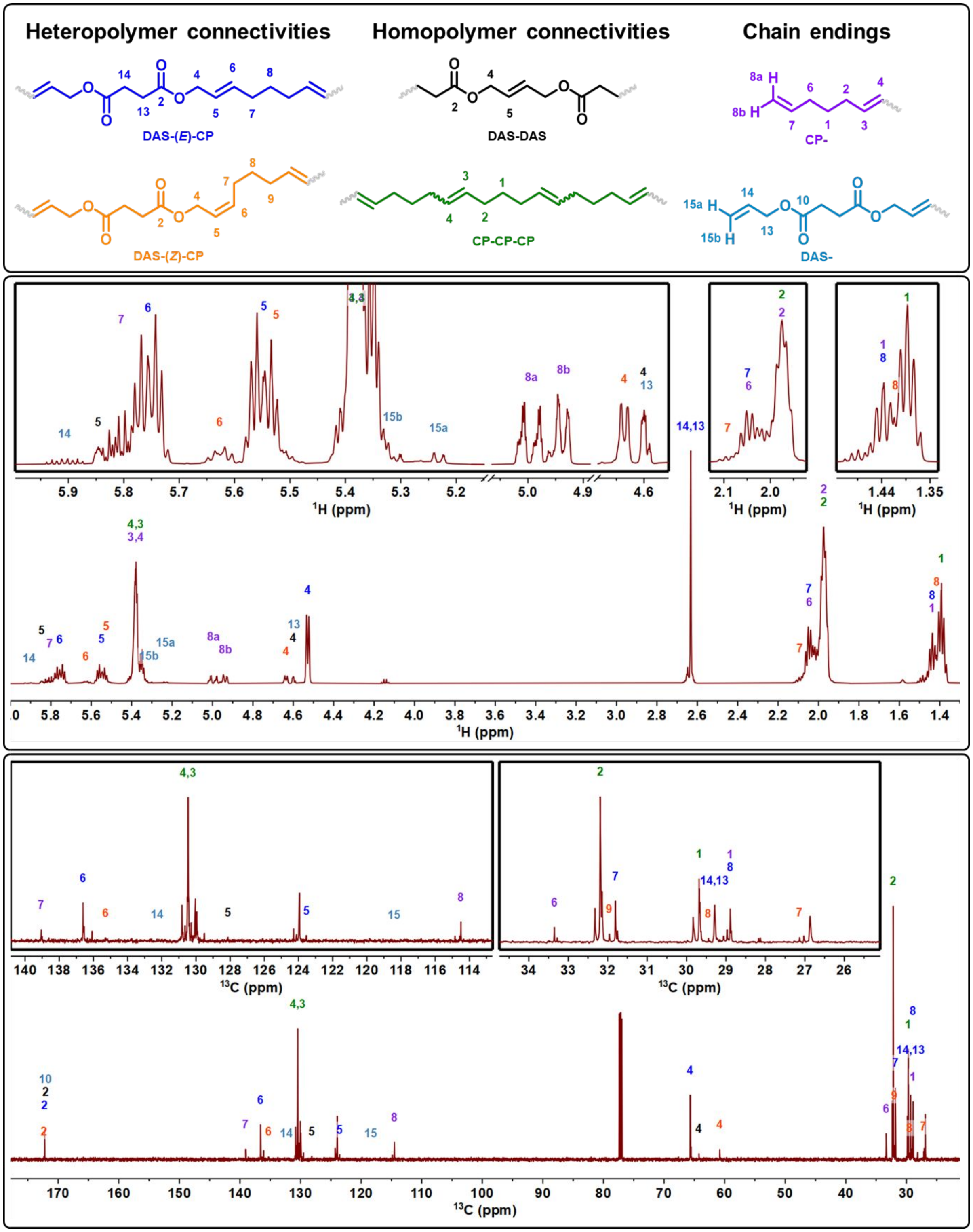

Figure S2. ¹H (middle panel) and ¹³C (lower panel) NMR spectra of copolymer ***poly*-(DAS-*co*-CP)-10**, illustrating the various detected moieties (upper panel).

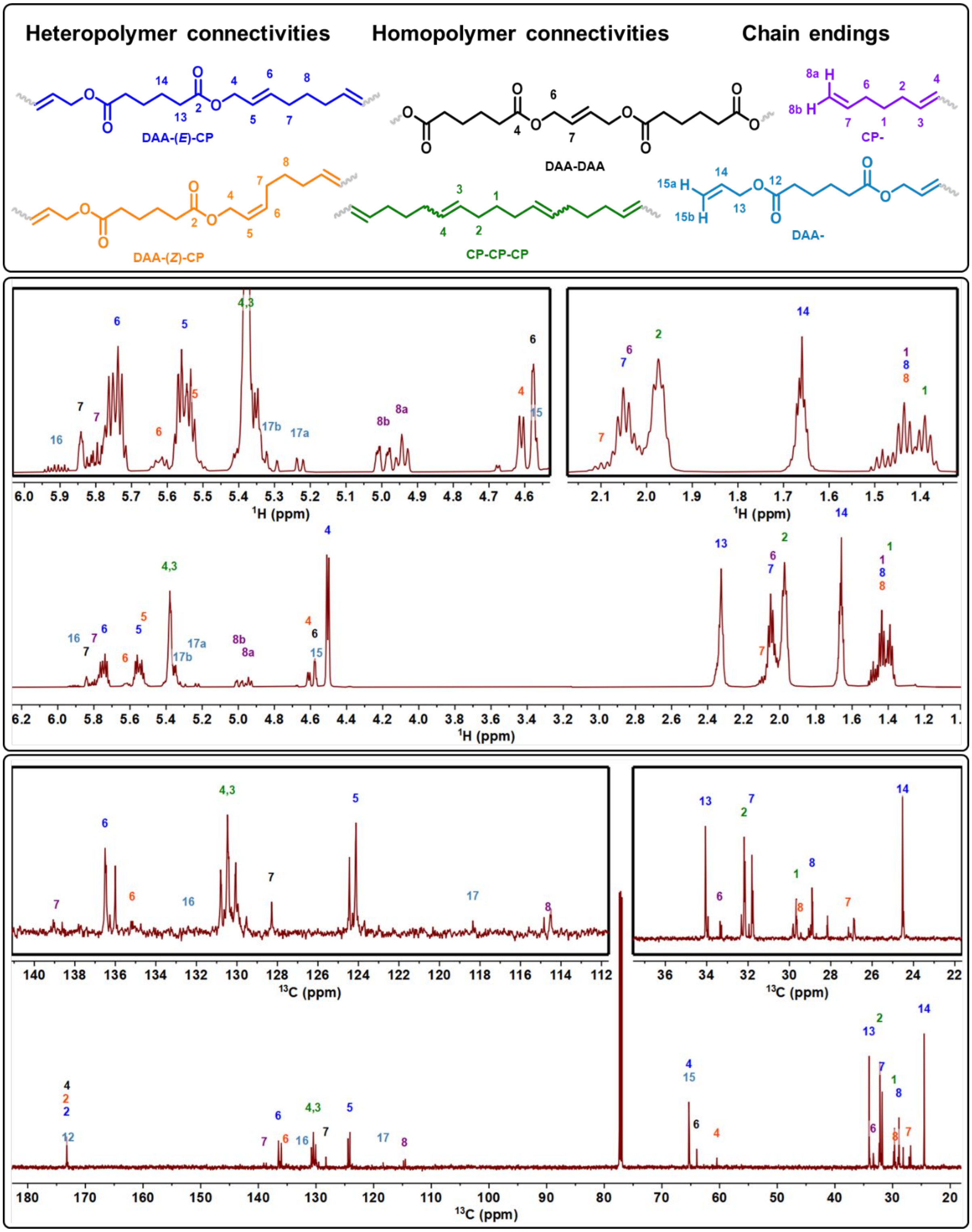

Figure S3. ^{1}H (middle panel) and ^{13}C (lower panel) NMR spectra of copolymer *poly*-**(DAA-*co*-CP)-10**, illustrating the various detected moieties (upper panel).

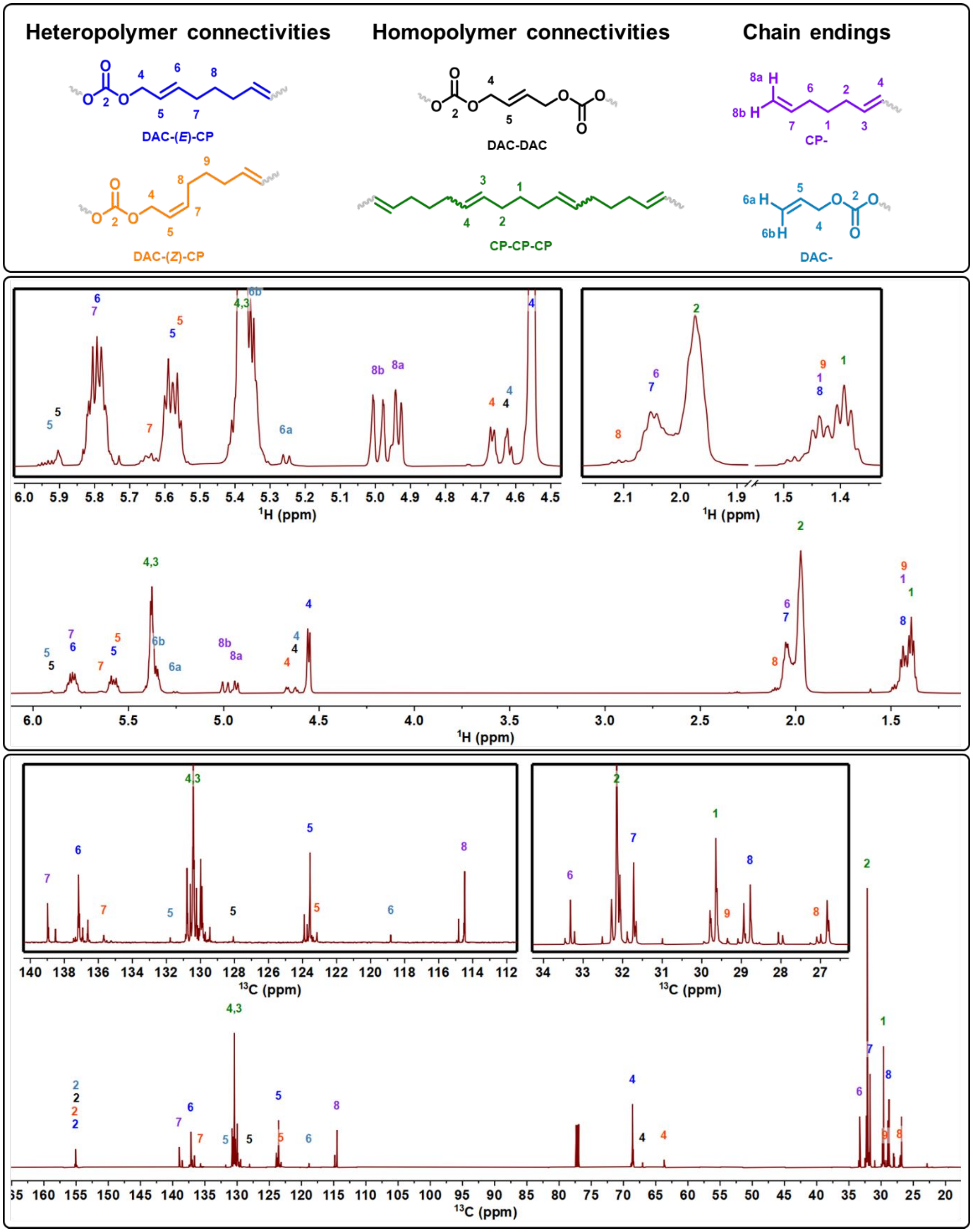

Figure S4. ^{1}H (middle panel) and ^{13}C (lower panel) NMR spectra of copolymer ***poly*-(DAC-*co*-CP)-10**, illustrating the various detected moieties (upper panel).

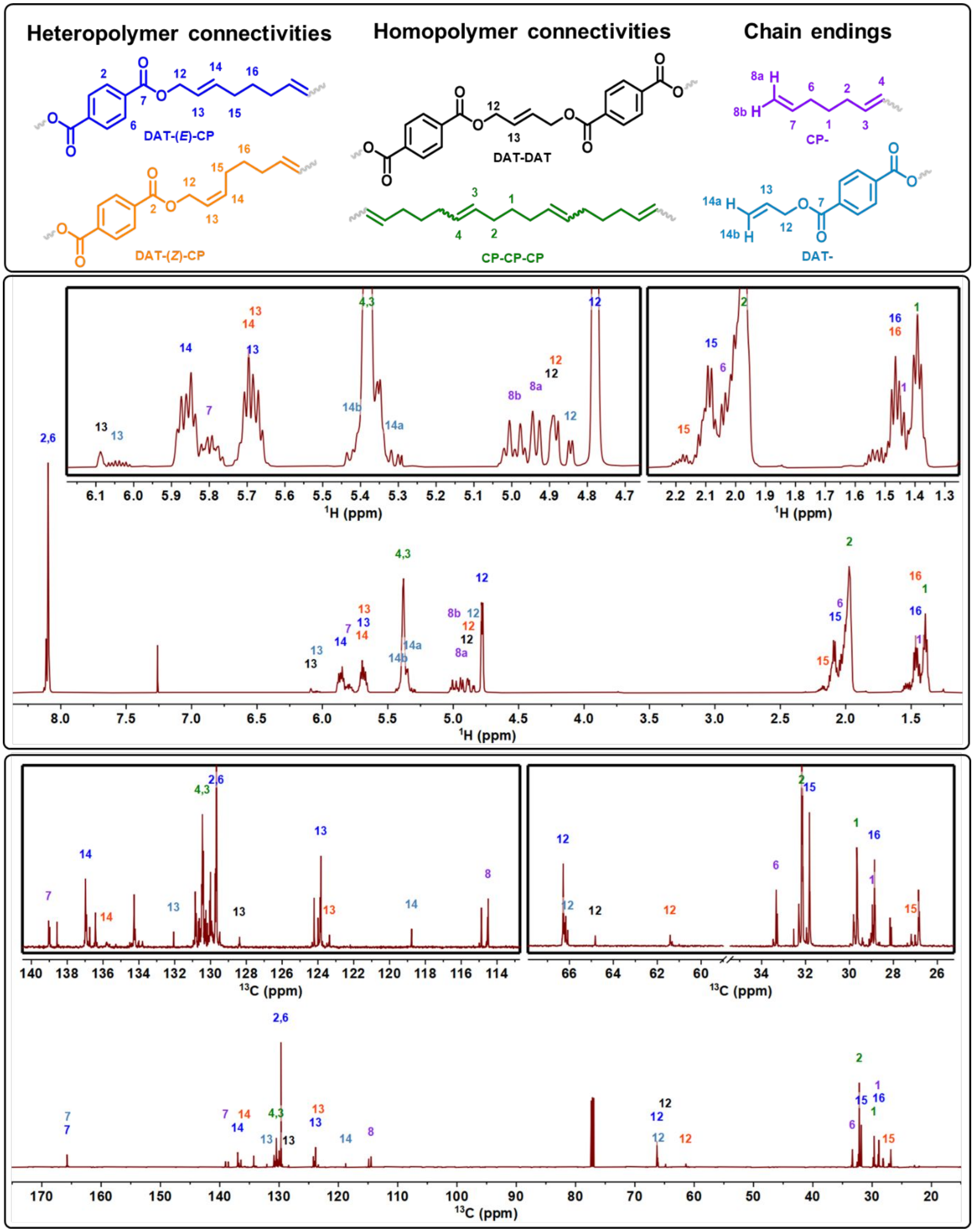

Figure S5. ^{1}H (middle panel) and ^{13}C (lower panel) NMR spectra of copolymer *poly*-**(DAT-*co*-CP)-10**, illustrating the various detected moieties (upper panel).

4. ADMET homopolymerization

4.1. Representative example of ADMET homopolymerization

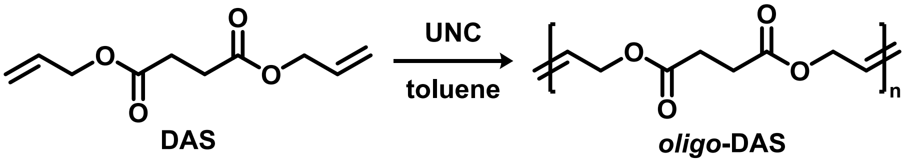

Diallyl succinate (500 mg, 2.5 mmol, 1 eq.) was placed in a vial and dissolved in 330 μL of toluene using a stirring bar, and toluene solution of **UNC** (3.7 mg, 2.5 μmol, 0.1 mol% in 170 μL toluene) was added. The mixture was stirred at RT for 3 hours. After that, the resulting precipitate was dissolved in THF, and ethyl vinyl ether (0.1 mL) was added. The mixture was stirred for 10 minutes, and MeOH was added to precipitate the polymer (350 mg, 82%).

4.2. NMR of ADMET homopolymers

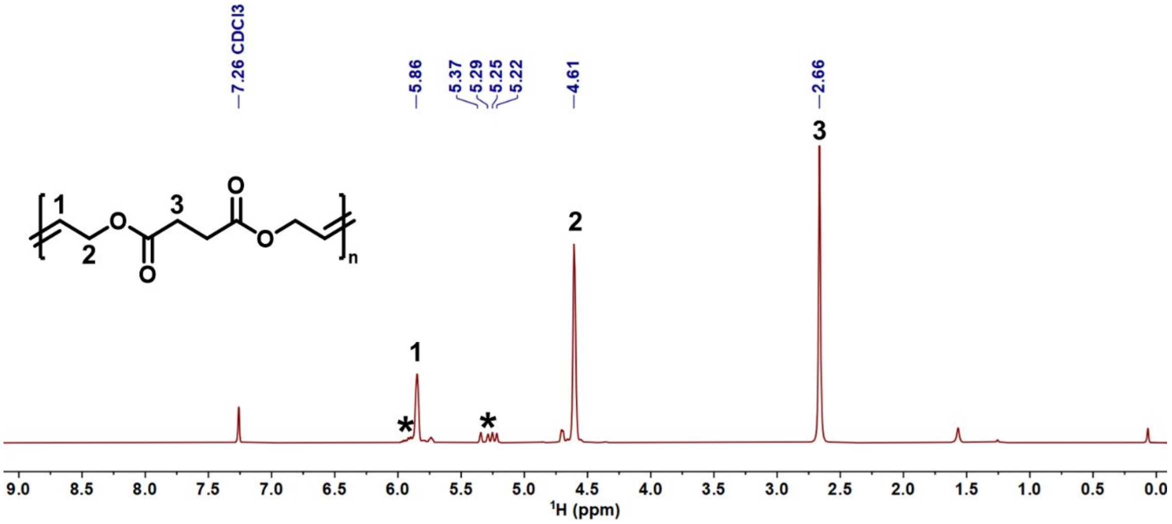

Figure S6. ^{1}H NMR spectra of *oligo*-**DAS** in $CDCl_3$. The chain-ending groups are marked with an asterisk.

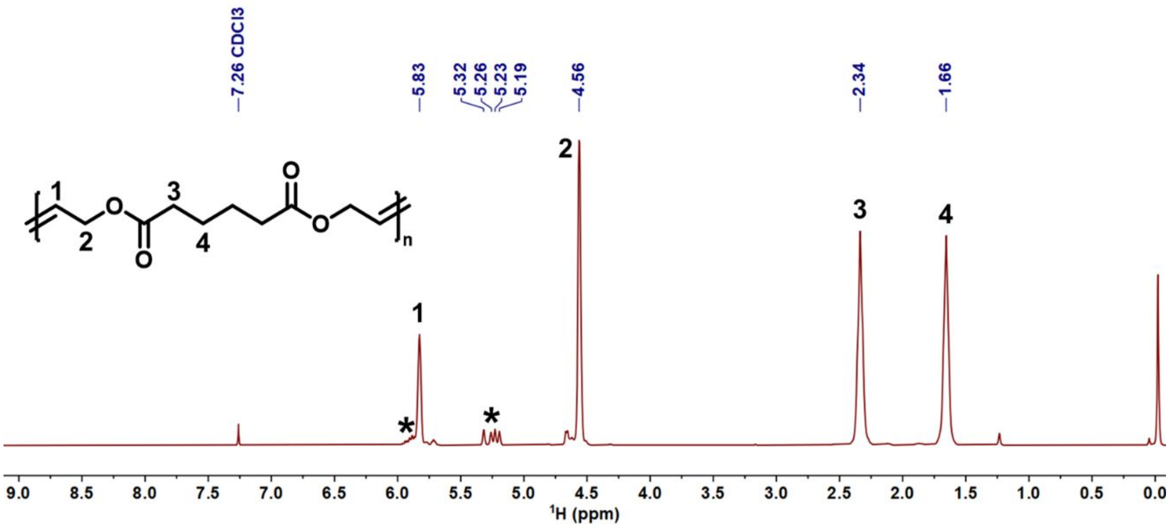

Figure S7. ^{1}H NMR spectra of *oligo*-**DAA** in $CDCl_3$. The chain-ending groups are marked with an asterisk.

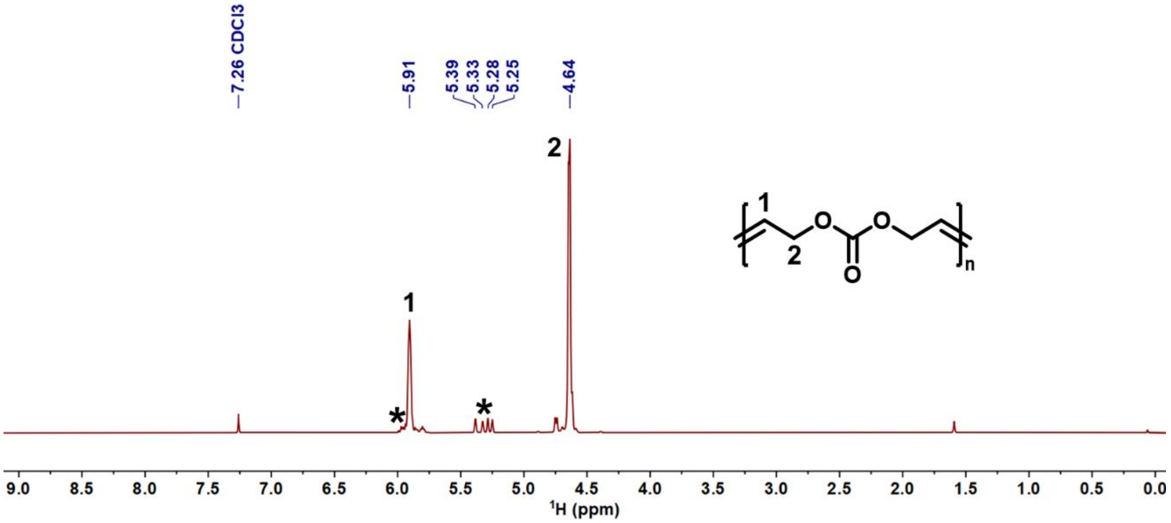

Figure S8. ^{1}H NMR spectra of *oligo*-**DAC** in $CDCl_3$. The chain-ending groups are marked with an asterisk.

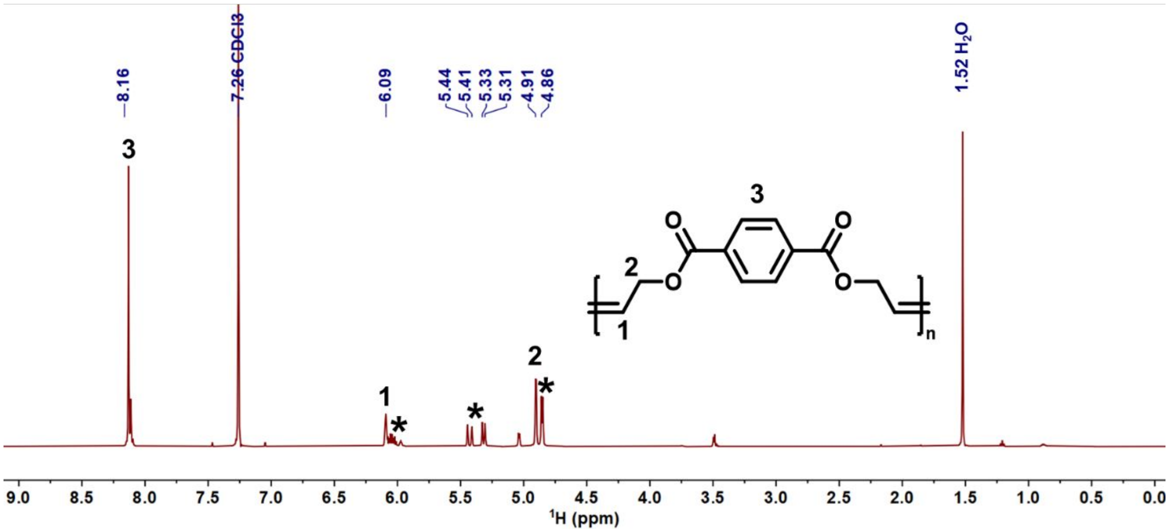

Figure S9. ^{1}H NMR spectra of *oligo*-**DAT** in $CDCl_3$. The chain-ending groups are marked with an asterisk.

5. ROIMP copolymerization

5.1. Representative example of ROIMP copolymerization in 10:1 ratio

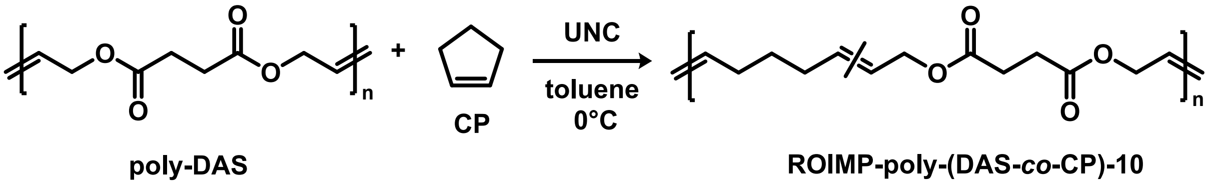

Cyclopentene (230 μL, 2.5 mmol) and *poly*-**DAS** (42 mg, 0.25 mmol diester unit) were placed in a vial with stirring bar, and were dissolved in toluene (0.95 mL), The solution was cooled to 0˚C and toluene solution of **UNC** (2.0 mg, 3.0 μmol, 0.1 mol% in 200 μL toluene) was added. The mixture was stirred at 0˚C for 3 hours. After that, the resulting precipitate was dissolved in THF, and ethyl vinyl ether (0.1 mL) was added. The mixture was stirred for 10 minutes, and MeOH was added to precipitate the polymer (90 mg, 41%).

5.2. Representative example of ROIMP copolymerization in 100:1 ratio

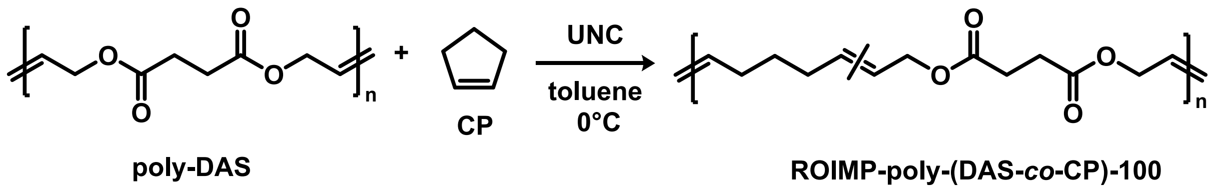

Cyclopentene (230 μL, 2.5 mmol) and *poly*-**DAS** (4.2 mg, 0.025 mmol diester unit) were placed in a vial with stirring bar and were dissolved in toluene (0.98 mL), The solution was cooled down to 0°C and toluene solution of **UNC** (1.7 mg, 2.5 μmol, 0.1 mol% in 170 μL toluene) was added. The mixture was stirred at 0°C for 3 hours. After that, the resulting precipitate was dissolved in THF, and ethyl vinyl ether (0.1mL) was added. The mixture was stirred for 10 minutes, and MeOH was added to precipitate the polymer (138 mg, 76%).

5.3. NMR analysis of ROIMP copolymerization

An example analysis of **ROIMP-** *poly*-(**DAS**-*co*-**CP**)-**10**:

The starting material *poly*-**DAS** has an approximate 6.4 degree of polymerization, based on amount of free chain ending groups. After the ROIMP reaction with 10 equivalents of cyclopentene, the formed polymer contains **DAS-DAS** and **DAS-CP** connectivities in an approximate ratio of 1:5, while the *poly*-**DAS** chain endings are fully converted to **DAS-CP** connectivities. This suggests that not only made the chain-ending terminal olefin groups react, but also the insertion of cyclopentene into the **DAS-DAS** chain occurred. If only the chain ending double bonds were reacting, then the ratio would be approximately 6:1 instead of 1:5.

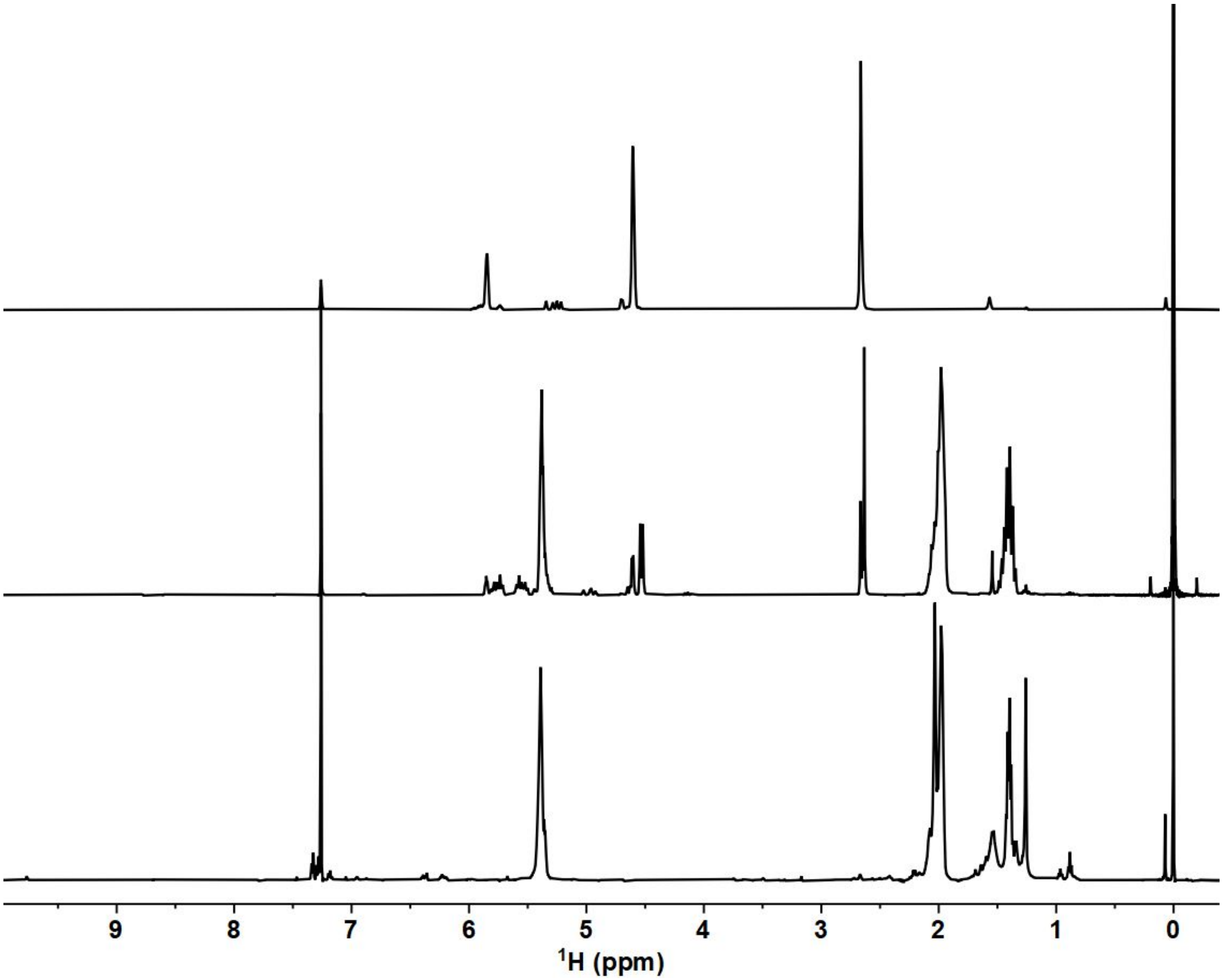

Figure S10. Representative example for ^{1}H NMR analysis of ROIMP polymerization. Stacked 1H NMR spectra of oligo-DAS oligomer (A), ROIMP-poly-(DAS-co-CP)-10, (B) and polypentenamer (PPe, C) ($CDCl_3$).

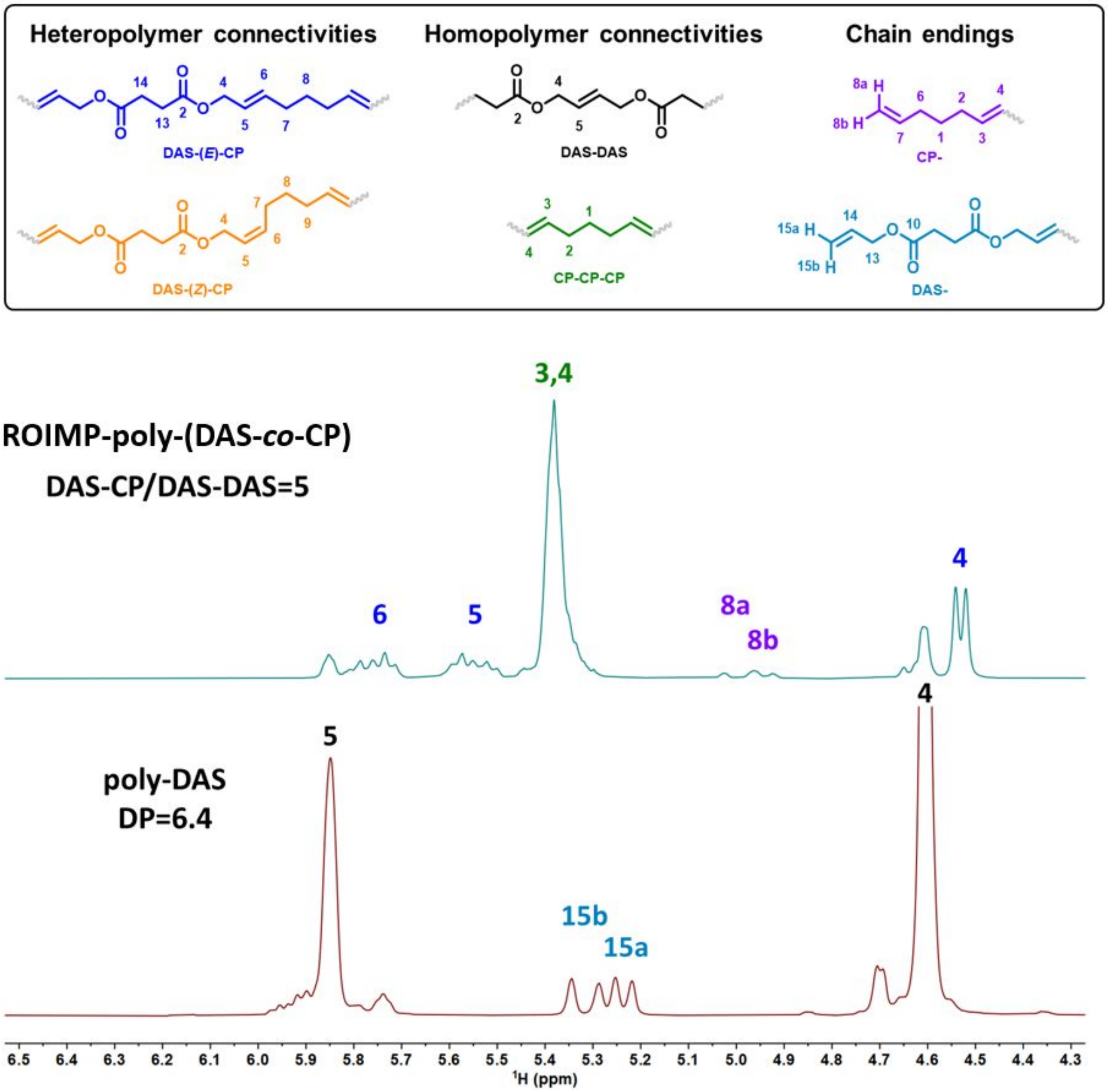

Figure S11. Stacked ¹H NMR spectra of ***poly*-DAS** and copolymer **ROIMP-*poly*-(DAS-*co*-CP)-10**, illustrating the various detected moieties (upper panel).

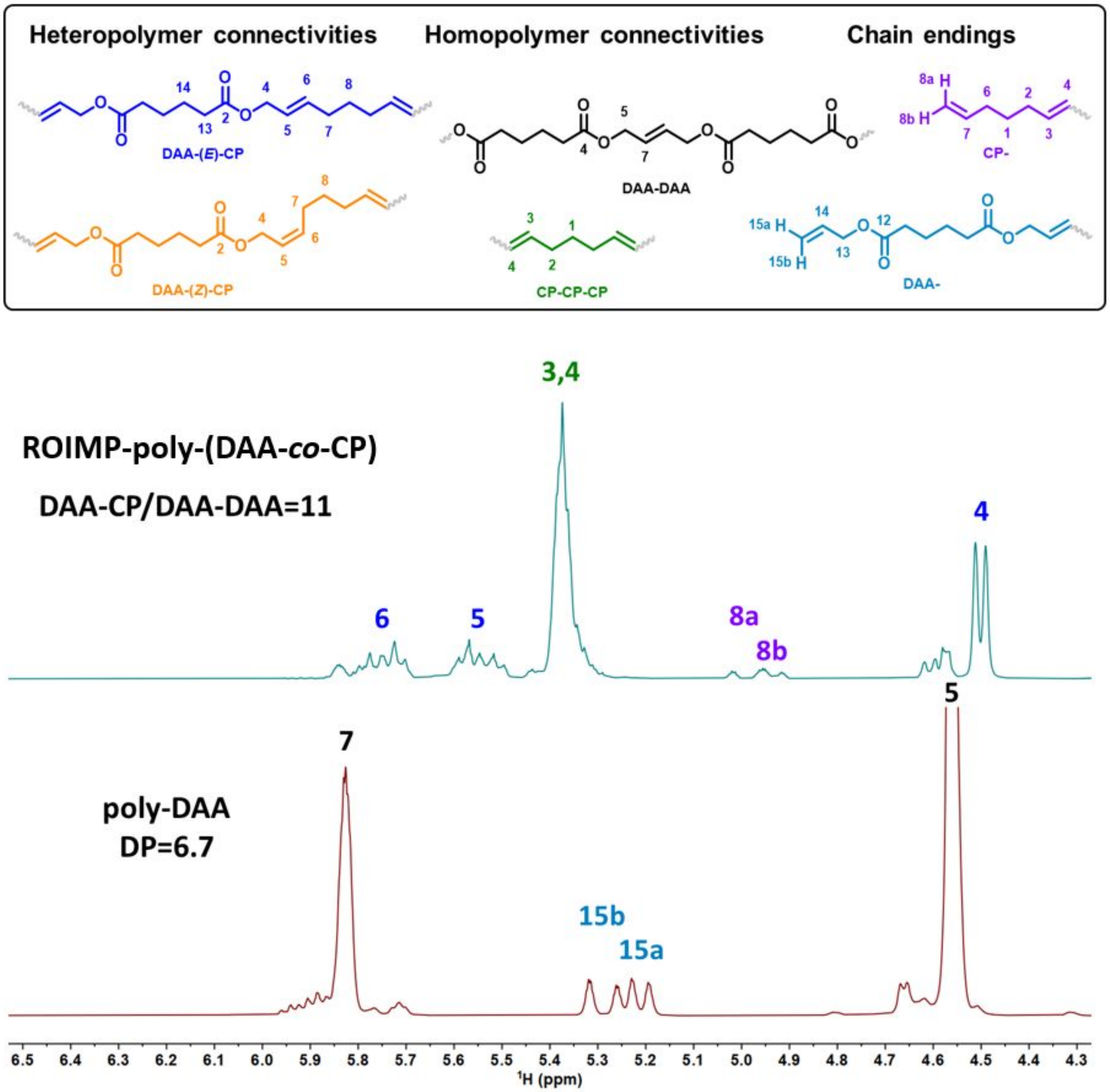

Figure S12. Stacked ^{1}H NMR spectra of *poly*-**DAA** and copolymer **ROIMP**-*poly*-**(DAA**-*co*-**CP)-10**, illustrating the various detected moieties (upper panel).

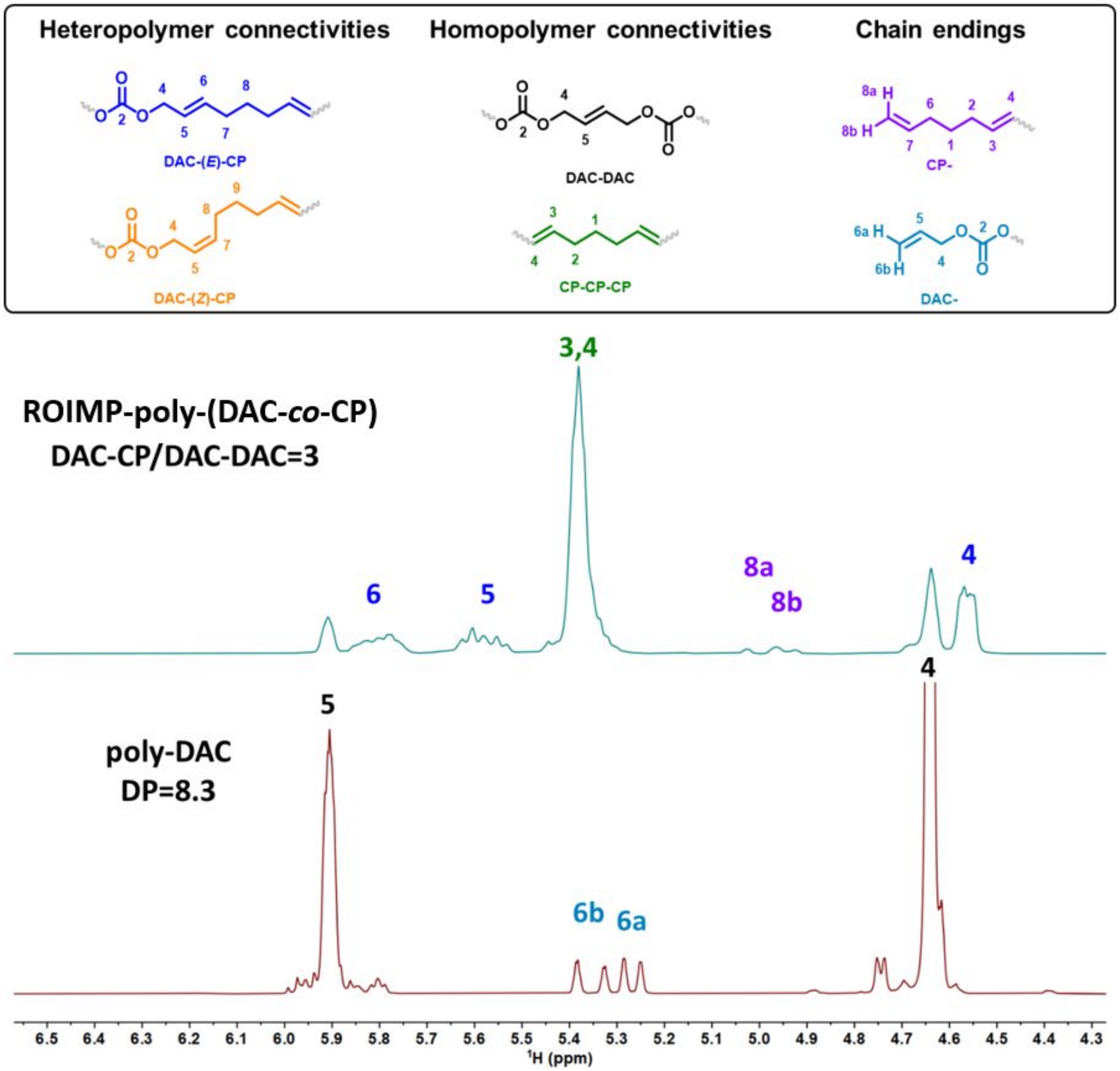

Figure S13. Stacked ¹H NMR spectra of *poly*-**DAC** and copolymer **ROIMP**-*poly*-(**DAC**-*co*-**CP**)-**10**, illustrating the various detected moieties (upper panel).

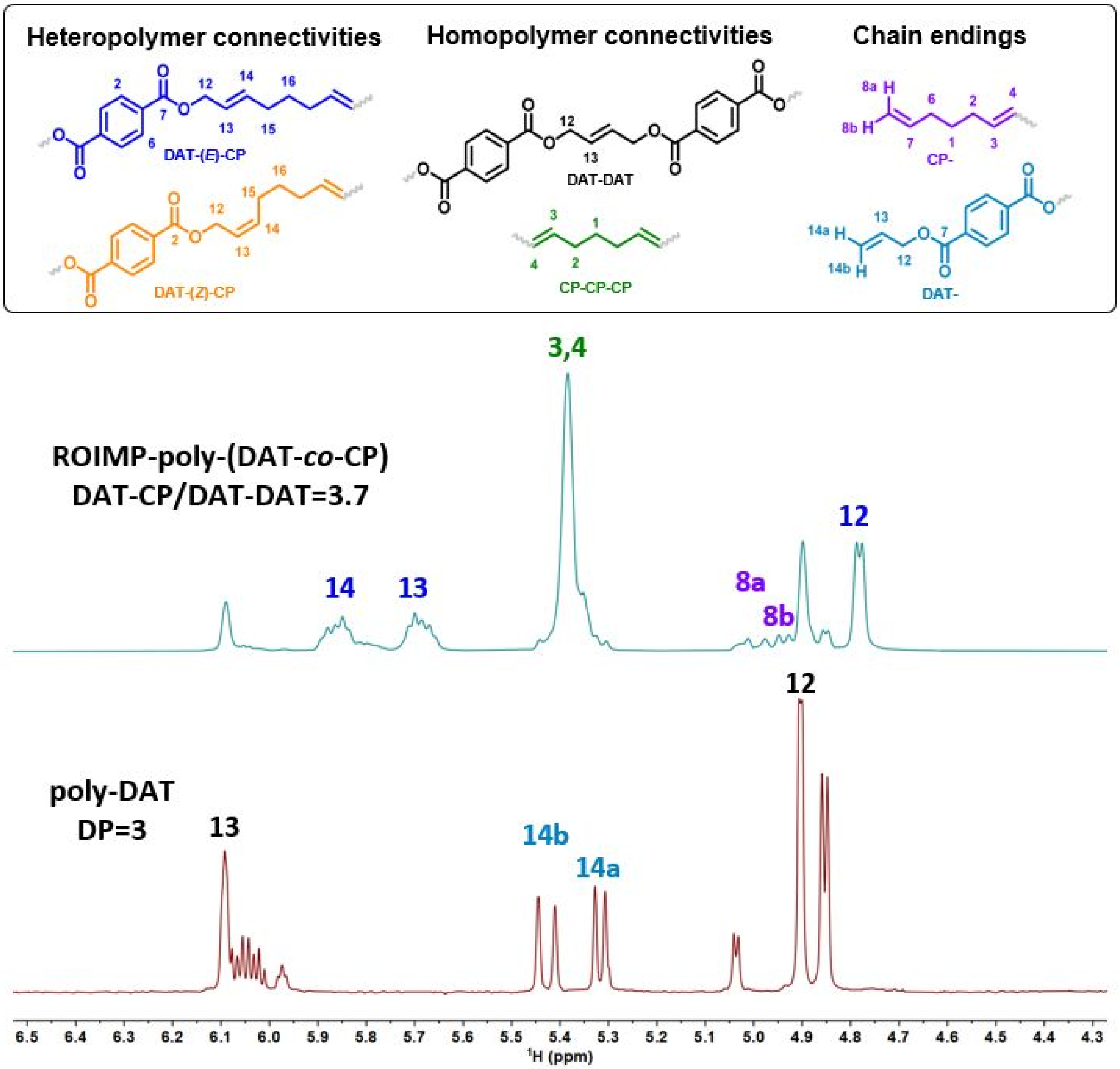

Figure S14. Stacked ^{1}H NMR spectra of *poly*-**DAT** and copolymer **ROIMP**-*poly*-**(DAT**-*co*-**CP)-10**, illustrating the various detected moieties (upper panel).

6. Hydrolysis

ROIMP-*poly*-(DAS-*co*-CP)-100 (50 mg) polymer was dissolved in THF (2 mL) and 2 M NaOH aqueous solution (4 mL) was added to the solution. The mixture was refluxed for 16 hours. After that the mixture was acidified with 5 M HCl, and the layers were separated, the aqueous layer was extracted with DCM (3 × 10 mL). The organic layers were combined, and the solvent was evaporated. The crude product was analyzed with APC (Figure S14). The conversion of the hydrolysis was determined by ^{1}H NMR through the integration of the methylene groups in the ester and allyl alcohol moieties (Figure S15).

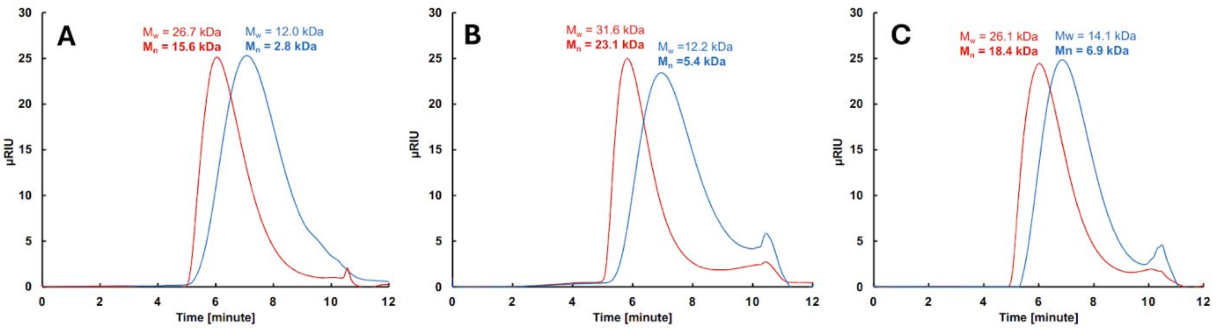

Figure S15. APC investigation of ROIMP-*poly*-(**DAS**-*co*-**CP**)-**100** (A), ROIMP-*poly*-(**DAA**-*co*-**CP**)-**100** (B) and ROIMP-*poly*-(**DAC**-*co*-**CP**)-**100** (C) polymers: before (red) and after hydrolysis (blue).

Table S1. Hydrolysis of ROIMP co-polymers. 50 mg polymer in THF (25 mg/mL), 4 mL 2 M NaOH, refluxed, t_r = 16 h.

Entry	Polymer (Table 5)	Conv. [%]	M_w[a] [kDa]	M_w[b] [kDa]	$Đ_M$[a] [-]	$Đ_M$[b] [-]
1	ROIMP-*poly*-(**DAS**-*co*-**CP**)-100	99	26.7	12.0	1.71	4.25
2	ROIMP-*poly*-(**DAA**-*co*-**CP**)-100	61	31.6	12.2	1.37	2.26
3	ROIMP-*poly*-(**DAC**-*co*-**CP**)-100	76	26.1	14.1	1.42	2.04

[a] before and [b] after polymer cleavage.

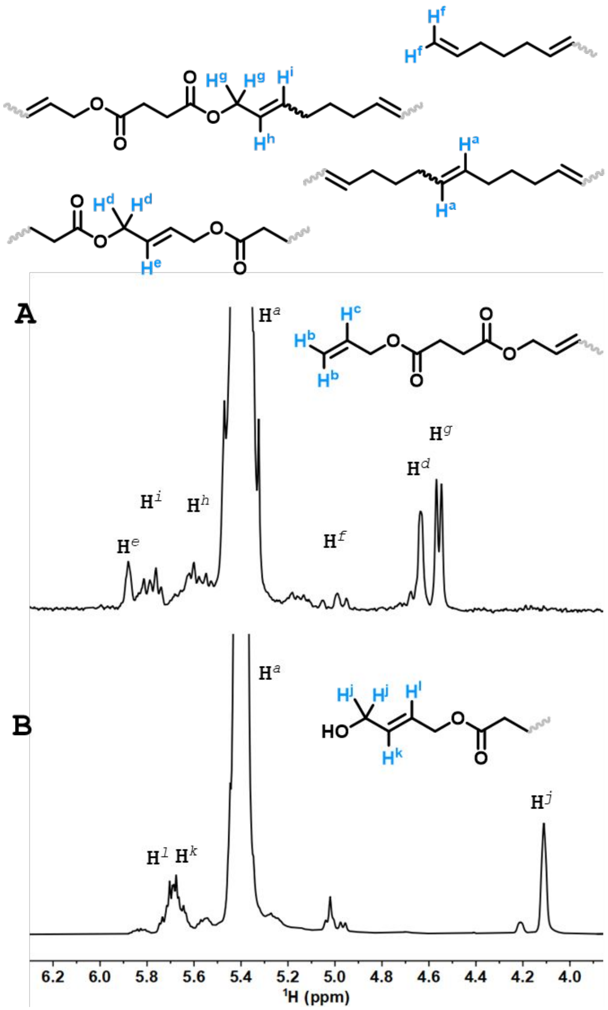

Figure S16. Stacked ¹H NMR spectra of ROIMP-*poly*-(**DAS**-*co*-**CP**)-**100** before (A) and after hydrolysis ($CDCl_3$) (B).

7. Hydrogenation of oligomers and ROIMP-copolymers

7.1. Representative example of hydrogenation of ROIMP-copolymers

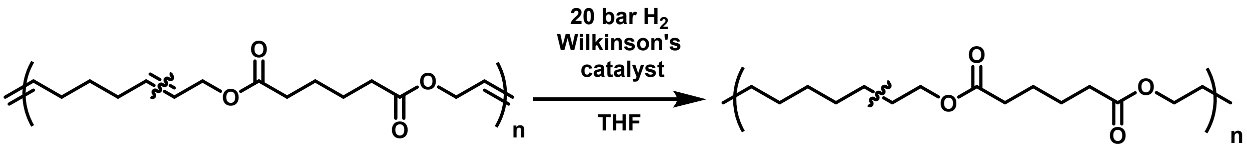

ROIMP-*poly*-(**DAS**-*co*-**CP**)-**10** (50 mg) and Wilkinson's catalyst (5 mg, 5 µmol) were dissolved in THF in a 20 mL autoclave. The autoclave was sealed, purged with hydrogen gas, and

pressurized to 20 bar. The reaction mixture was stirred at 100 ˚C for 16 hours. Subsequently, the pressure was released, and the insoluble product was filtered out (46 mg, 90%).

Table S2. Polymerization degree of ADMET oligomers before and after hydrogenation. 50 mg polymer in THF (25 mg/mL), Wilkinson's catalyst (1 mg, 1 µmol), 20 bar H_2, 50 ˚C, t_r = 16 h.

Entry	Polymer (Table 2)	DP[a]	DP[b]
1	*poly*-**DAS**	6.4	5.8
2	*poly*-**DAA**	6.7	5.4
3	*poly*-**DAC**	8.3	8.0

[a] before and [b] after hydrogenation. Degree of polymerization (DP) is determined by ^1H NMR.

Table S3. Yield of formation and thermal analysis data for hydrogenated ROIMP co-polymers.

Entry	Polymer (Table 5)	Isolated yield [%]	T_m [˚C]	T_d [˚C]
1	ROIMP-*poly*-(**DAS**-*co*-**CP**)-100-H_2	99	110	410
2	ROIMP-*poly*-(**DAA**-*co*-**CP**)-100-H_2	61	111	400
3	ROIMP-*poly*-(**DAC**-*co*-**CP**)-100-H_2	76	107	435

7.2. NMR analysis of hydrogenated oligomers

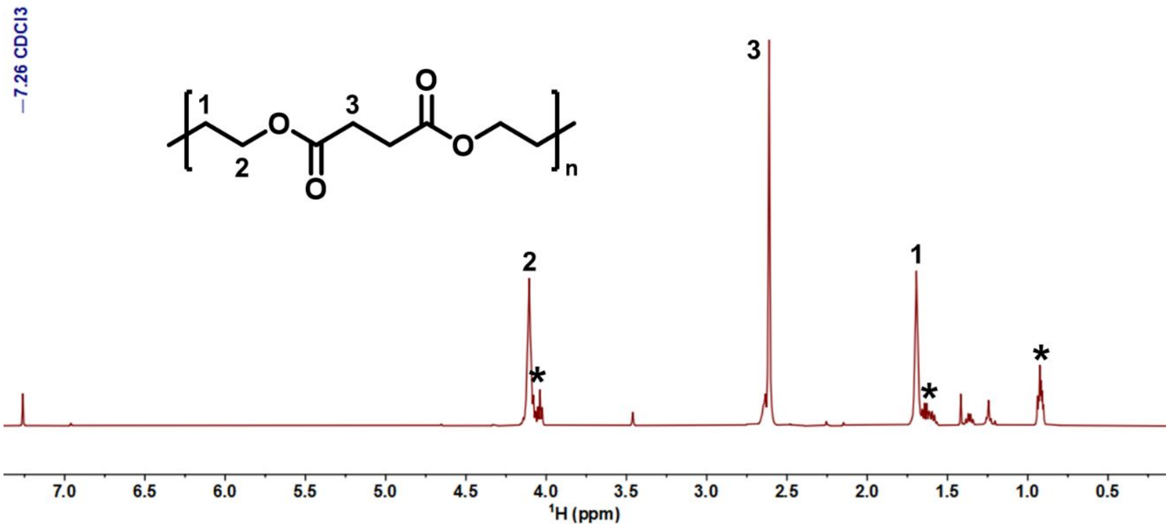

Figure S17. ^{1}H NMR spectra of hydrogenated *poly*-**(DAS)** in $CDCl_3$. The -CH_2- moiety of the newly formed polyethylene chain is marked with an asterisk.

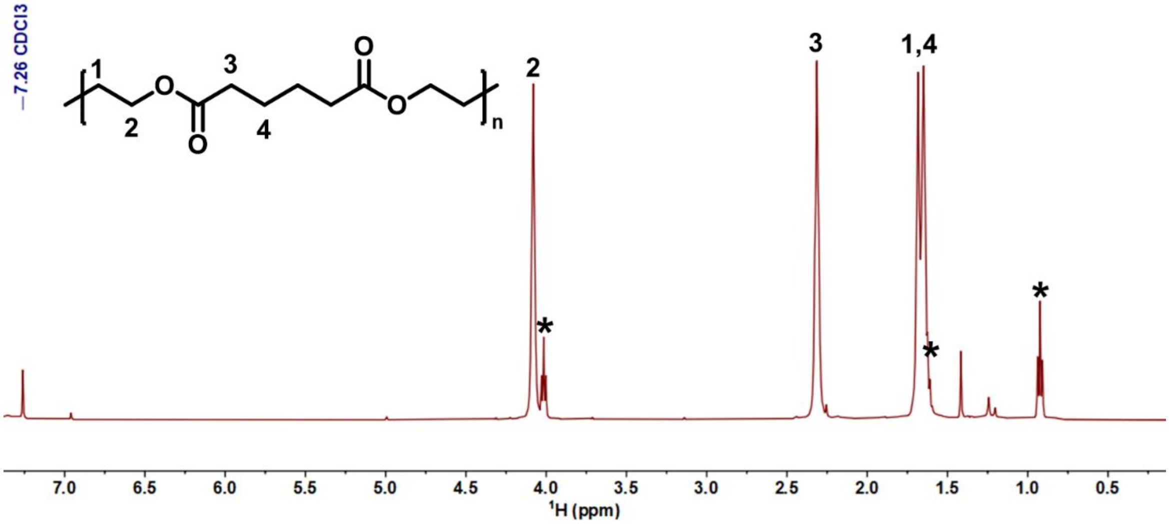

Figure S18. ^{1}H NMR spectra of hydrogenated *poly*-**(DAA)** in $CDCl_3$. The -CH_2- moiety of the newly formed polyethylene chain is marked with an asterisk.

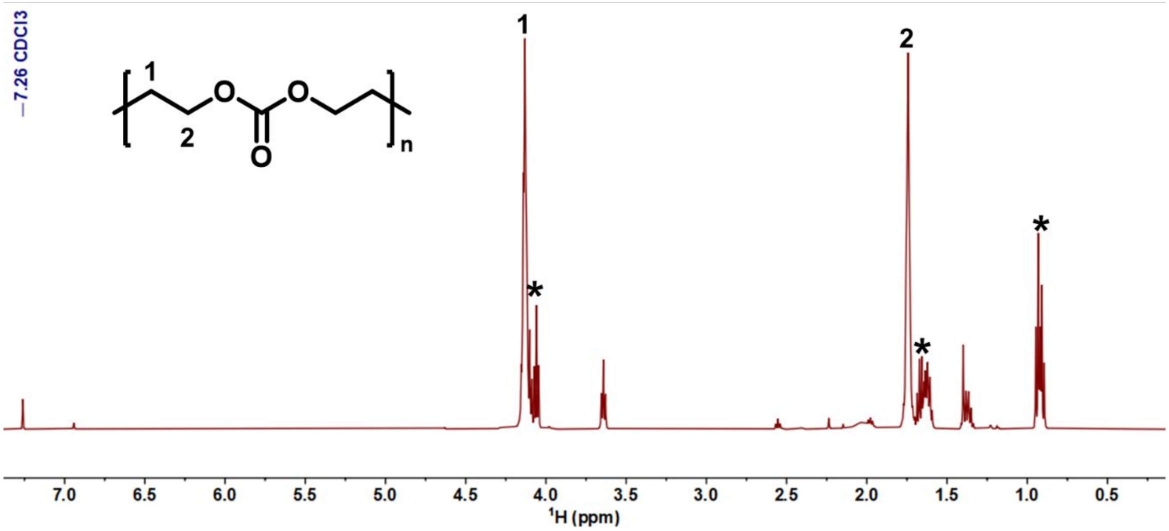

Figure S19. ^{1}H NMR spectra of hydrogenated *poly*-**(DAC)** in $CDCl_3$. The $-CH_2-$ moiety of the newly formed polyethylene chain is marked with an asterisk.

7.3. NMR analysis of hydrogenated ROIMP-copolymers

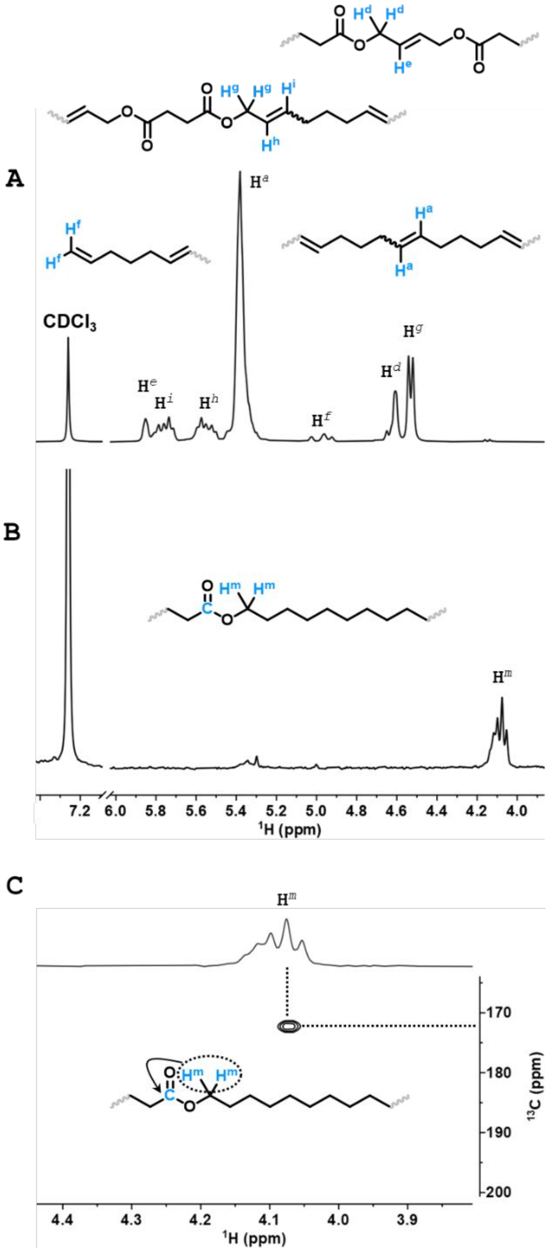

Figure S20. Stacked ^{1}H NMR spectra of ROIMP-*poly*-(**DAS**-*co*-**CP**)-**10** before (A) and after (B) hydrogenation in $CDCl_3$. ^1H – ^{13}C HMBC spectra of the hydrogenated product, showing correlation between carbonyl groups and the -O-CH_2- moiety (C). For H^h and H^f assignation see Figure 1; 50 mg polymer in THF (25 mg/mL), Wilkinson's catalyst (1 mg, 1 µmol), 20 bar H_2, 50 ˚C, t_r = 16 h.

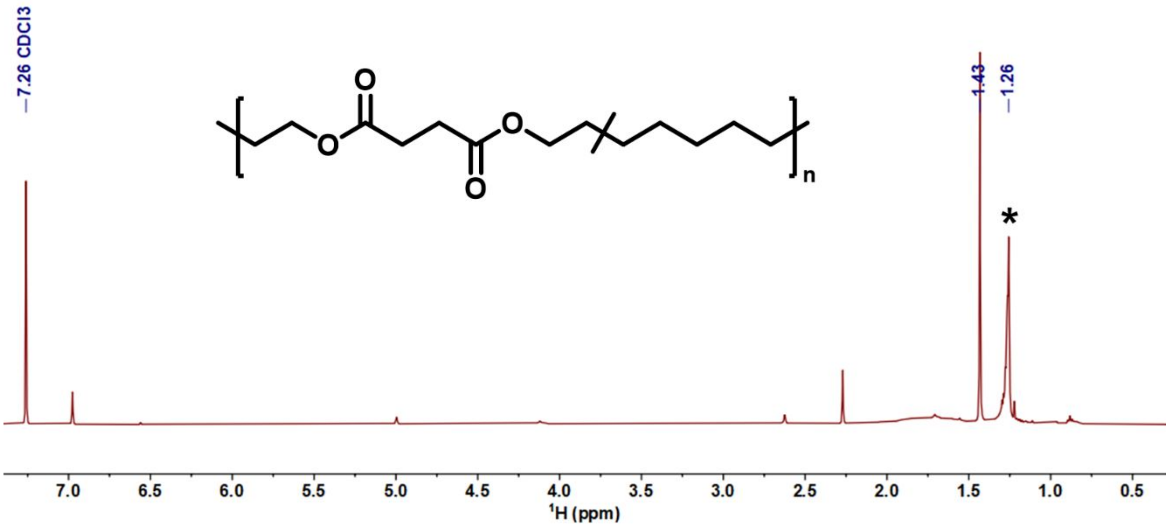

Figure S21. ^{1}H NMR spectra of hydrogenated **ROIMP**-*poly*-**(DAS**-co-**CP)-100** in $CDCl_3$. The -CH_2- moiety of the newly formed polyethylene chain is marked with an asterisk.

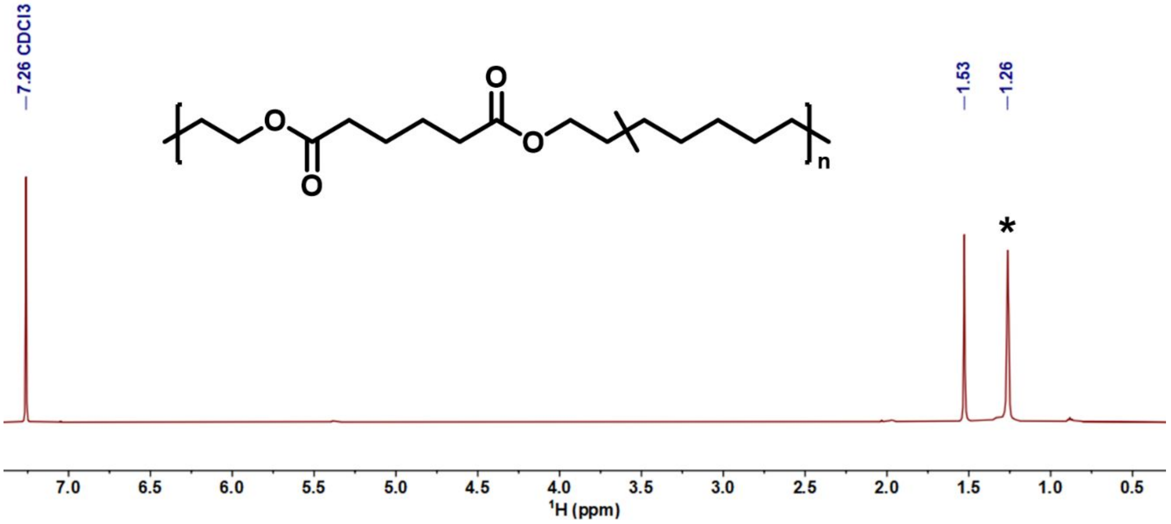

Figure S22. ^{1}H NMR spectra of hydrogenated **ROIMP**-*poly*-**(DAA**-*co*-**CP)-100** in $CDCl_3$. The -CH_2- moiety of the newly formed polyethylene chain is marked with an asterisk.

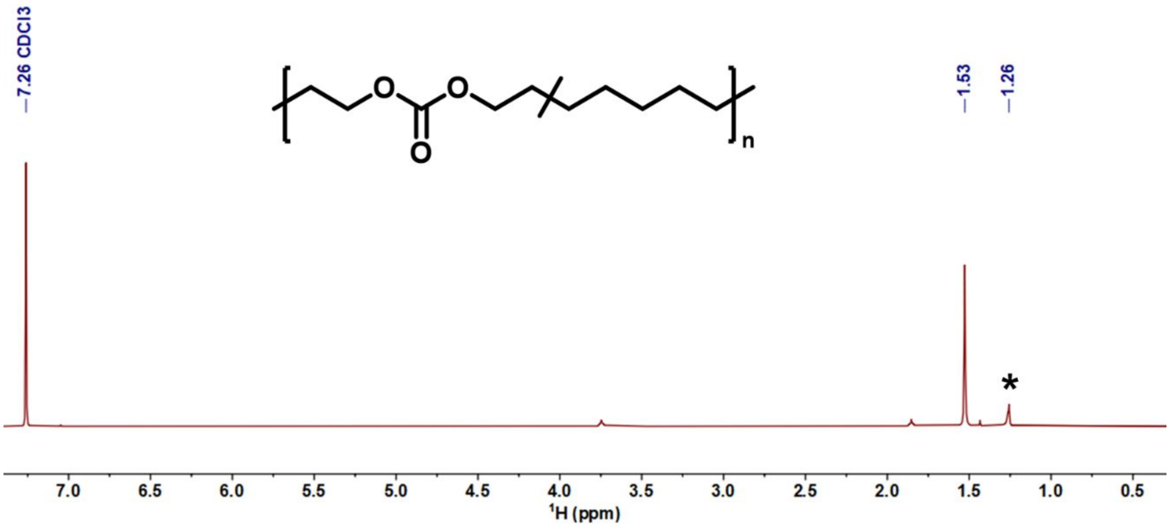

Figure S23. ^{1}H NMR spectra of hydrogenated **ROIMP-*poly*-(DAC-*co*-CP)-100** in $CDCl_3$. The -CH_2- moiety of the newly formed polyethylene chain is marked with an asterisk.

8. Representative example for comparison of TGA diagrams before and after hydrogenation.

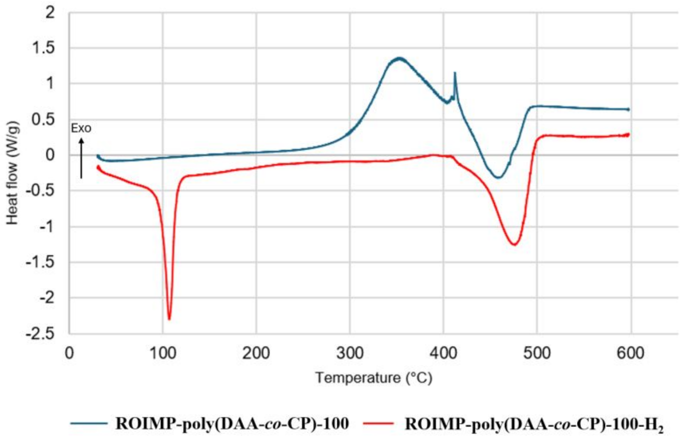

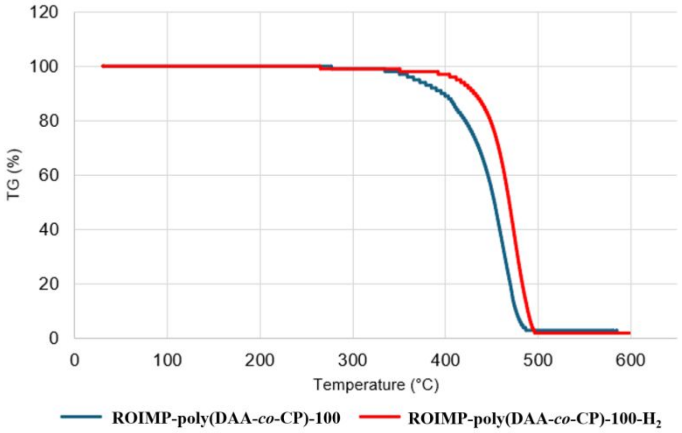

Figure S24. Representative example of DSC (A) and TGA (B) data for **ROIMP**-*poly*-(**DAA**-*co*-**CP**)-100 and **ROIMP**-*poly*-(**DAA**-*co*-**CP**)-100-H_2.

9. Scaled-up synthesis of ROIMP-*poly*-(DAS-*co*-CP)-100 and ROIMP-*poly*-(DAS-*co*-CP)-200

9.1. Scaled-up ADMET homopolymerization

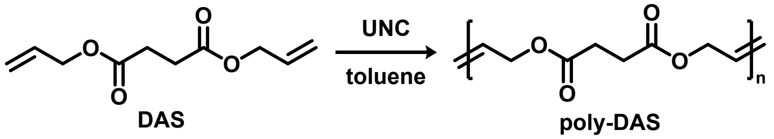

Diallyl succinate (3.1 g, 15.6 mmol, 1 eq.) was placed in a vial and dissolved in 3.1 mL of toluene with stirring bar and toluene solution of **UNC** (23.1 mg, 15.6 μmol, 0.1 mol%) was added. The mixture was stirred at RT for 3 hours. After that, the resulting precipitate was dissolved in THF, and ethyl vinyl ether (1.0 mL) was added. The mixture was stirred for 10 minutes, and MeOH was added to precipitate the polymer (1.5 g, 56 %).

9.2. Scaled-up ROIMP copolymerization of poly-DAS in 100:1 ratio

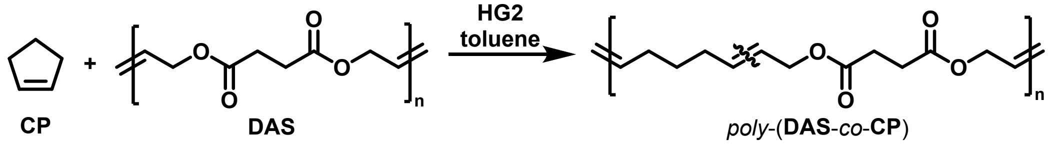

Cyclopentene (40 mL, 435 mmol) and *poly*-**DAS** (0.73 g, 4.35 mmol diester unit) were placed in a vial with stirring bar and were dissolved in toluene (160 mL), The solution was cooled to 0°C and toluene solution of **HG2** (325.8 mg, 0.52 mmol, 0.1 mol%) was added. The mixture was stirred at 0°C for 24 hours. After that, the resulting precipitate was dissolved in THF, and ethyl vinyl ether (1.0 mL) was added. The mixture was stirred for 10 minutes, and MeOH was added to precipitate the polymer (16.0 g, 64%).

NMR analysis of the polymer indicated a **CP–CP** to **CP–DAS** moiety ratio of 40:1.

APC measurements showed a M_w of 22.5 kDa, and thus a second ROIMP reaction was carried out as follows.

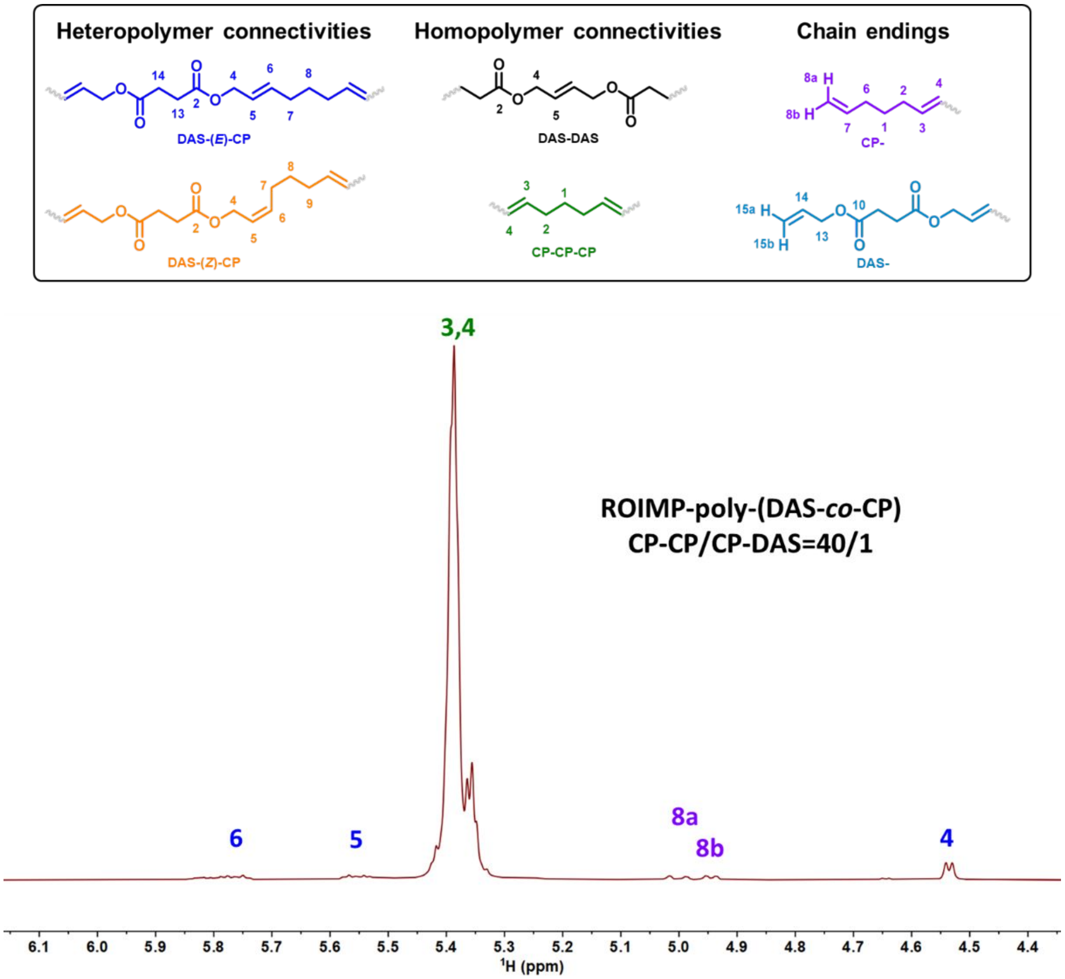

Figure S25. ^{1}H NMR spectra of scaled up **ROIMP**-*poly*-**(DAC**-*co*-**CP)-100** in $CDCl_3$. Indicating the ratio of the connected **CP-CP** and **CP-DAS** moieties.

9.3. Scaled-up ROIMP copolymerization of ROIMP-*poly*-(DAS-co-CP)-100

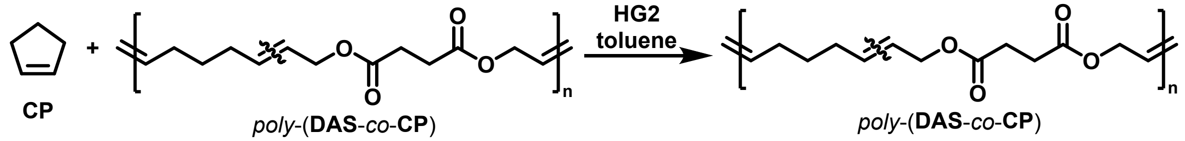

Cyclopentene (40 mL, 435 mmol) and ROIMP-*poly*-(**DAS-co-CP)-100** (16.0 g) were placed in a vial with stirring bar and were dissolved in toluene (150 mL), The solution was cooled to 0˚C **HG2** (281 mg, 0.45 mmol, 0.1 mol%) was added. The mixture was stirred at 0˚C for 24 hours. After that, the resulting precipitate was dissolved in THF, and ethyl vinyl ether (1.0 mL) was added. The mixture was stirred for 10 minutes, and MeOH was added to precipitate the polymer.

The product was washed with additional MeOH (2x100 mL) to yield the chain-extended polymer (34.6 g, 56%).

NMR analysis of the polymer indicated a **CP–CP** to **CP–DAS** moiety ratio of 100:1.

APC measurements showed a M_w of 27.3 kDa for the polymer.

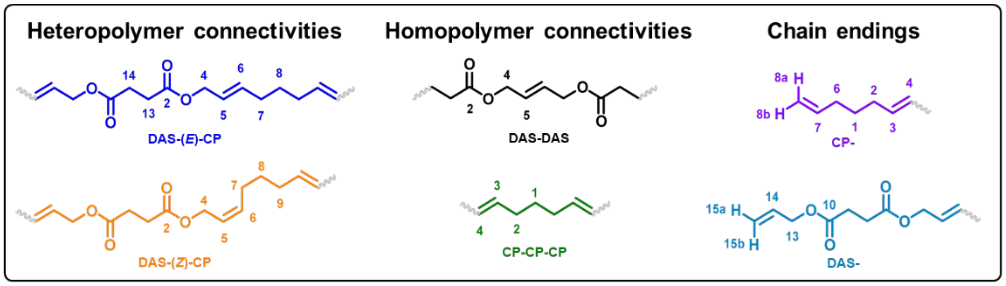

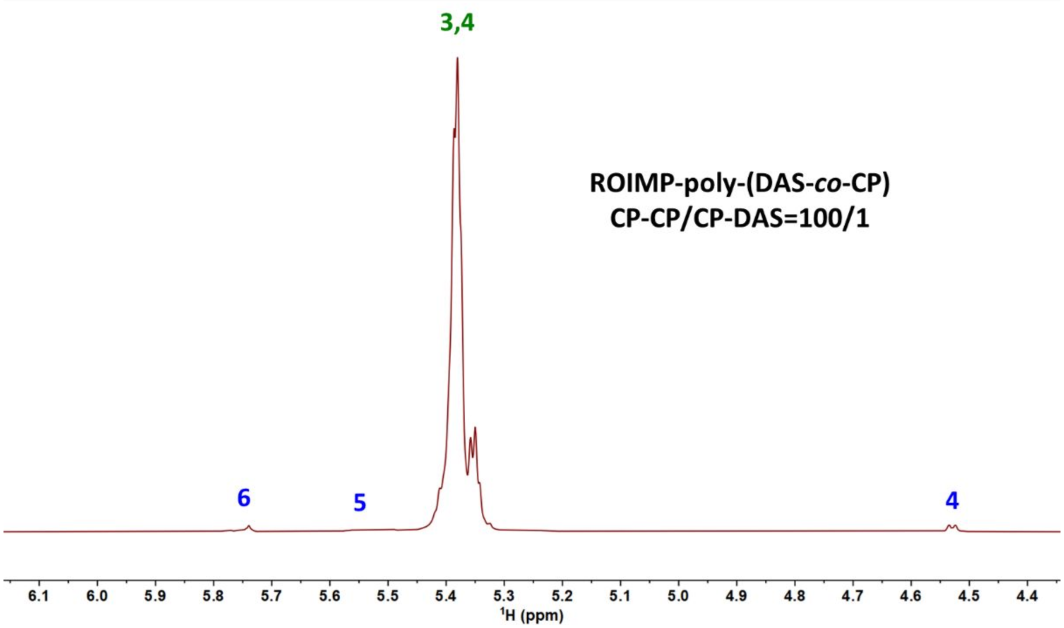

Figure S26. ^{1}H NMR spectra of the second scaled up **ROIMP-*poly*-(DAS-*co*-CP)-200** in $CDCl_3$. Indicating the ratio of the connected **CP-CP** and **CP-DAS** moieties.

9.4. Scaled-up hydrogenation of ROIMP-*poly*-(DAS-*co*-CP)-200

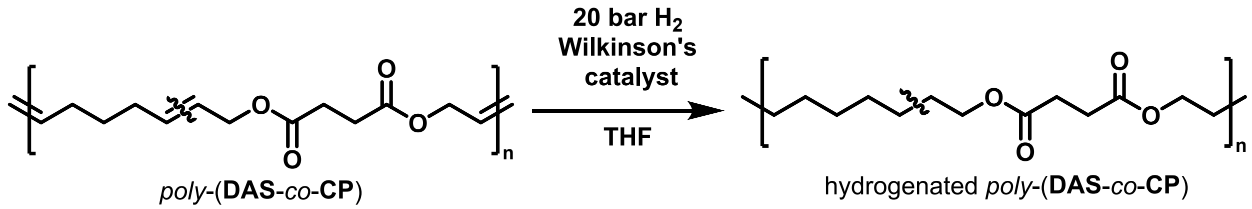

ROIMP-*poly*-(DAS-*co*-CP)-200 (8.0 g) and Wilkinson's catalyst (160 mg, 0.173 mmol) were dissolved in THF (300 mL). The autoclave was sealed, purged with hydrogen gas, and pressurized to 20 bar. The reaction mixture was stirred at 50 ˚C for 48 hours, and occasionally repressurized. 250 mL MeOH was added to the suspension, and after 15 minutes of stirring, it was filtered and washed fresh MeOH (3 × 250 mL) to yield the polymer as a white solid (6.2 g, 78%).

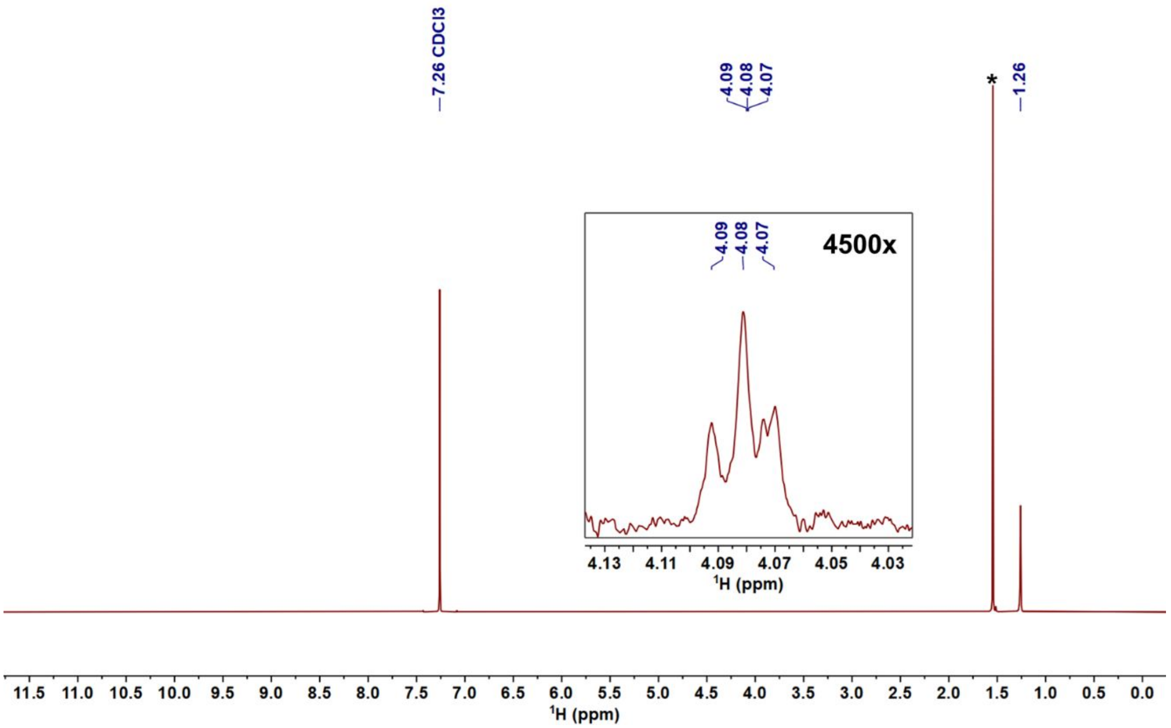

Figure S27. ^{1}H NMR spectra of the hydrogenated **ROIMP-*poly*-(DAS-*co*-CP)-200** in $CDCl_3$. The ester –O-CH2- group is expanded for clarity at 4.08 ppm. Asterisk indicates water.

10. Physical properties

Standard specimens for mechanical characterization (ISO 527/2 5A) were produced by compression molding (Fontijne SRA100) at 150 ˚C mold temperature, 150 kN pressing force, and a 5-minute holding time. All specimens were kept in a room with controlled temperature and humidity (23 ˚C and 50%) for at least one week prior to further testing. Mechanical properties were characterized by tensile testing on standard 4 mm thick ISO 527/2 5A specimens using an Instron 5566 apparatus (Instron, Norwood, MA, USA). Stiffness (E) was determined at 0.5 mm/min cross-head speed and 50 mm gauge length. Tensile strength (σ) and elongation-at-break (ε) were calculated from force vs. deformation traces measured on the same specimens at 10 mm/min cross-head speed.

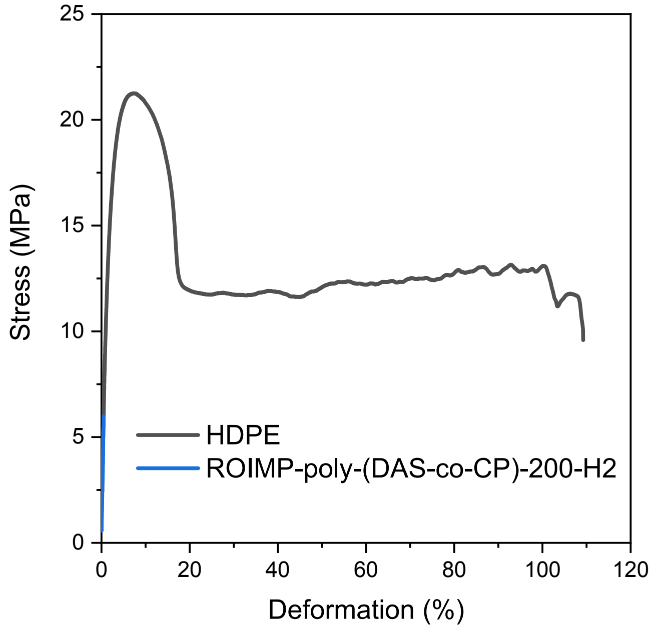

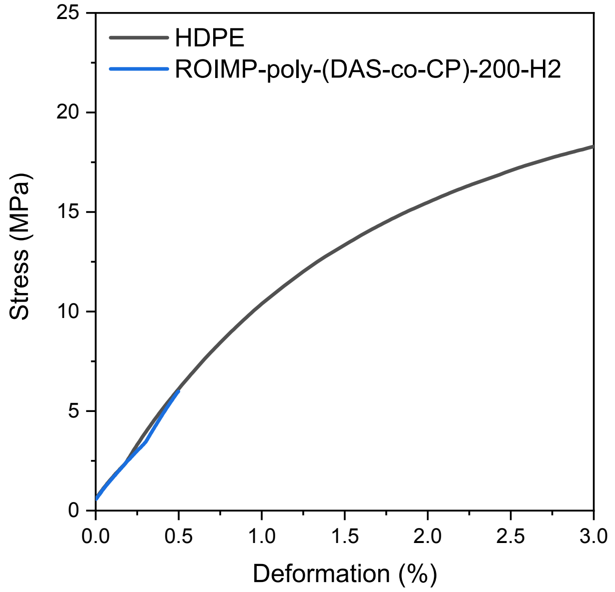

Figure S27. Stress-strain curves of **ROIMP**-poly-(**DAS**-co-**CP**)-200-H_2 (top) and magnification of the initial part (bottom)

Thermal properties were determined by differential scanning calorimetry using a Perkin Elmer DSC 7 (PerkinElmer, Inc., CT, USA) apparatus. Two heating and one cooling run were done on

5 mg samples with a heating rate of 10 ˚C/min between room temperature and 160 ˚C. The purge gas was pure nitrogen.

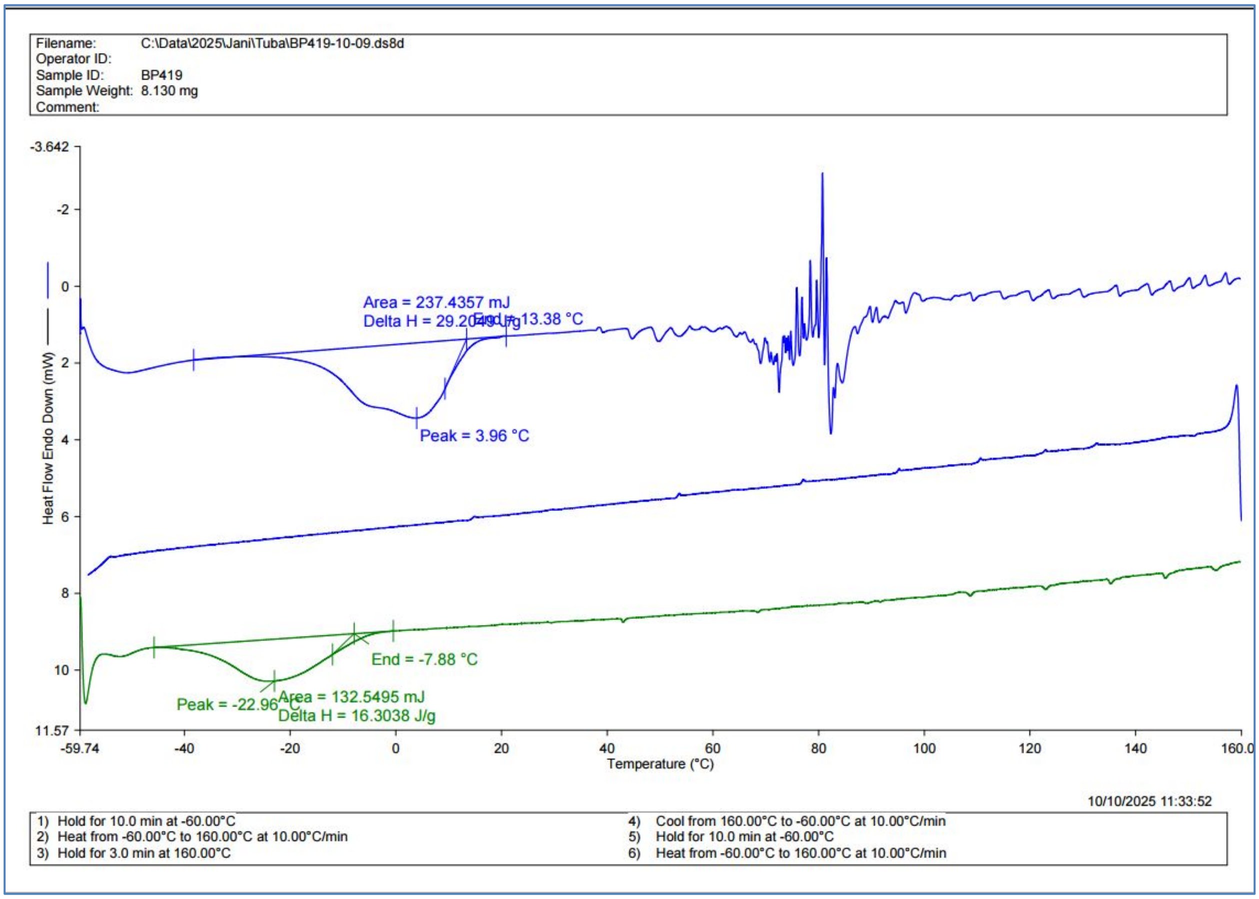

Figure S28. DSC traces of the **ROIMP-*poly*-(DAS-*co*-CP)-200** sample.

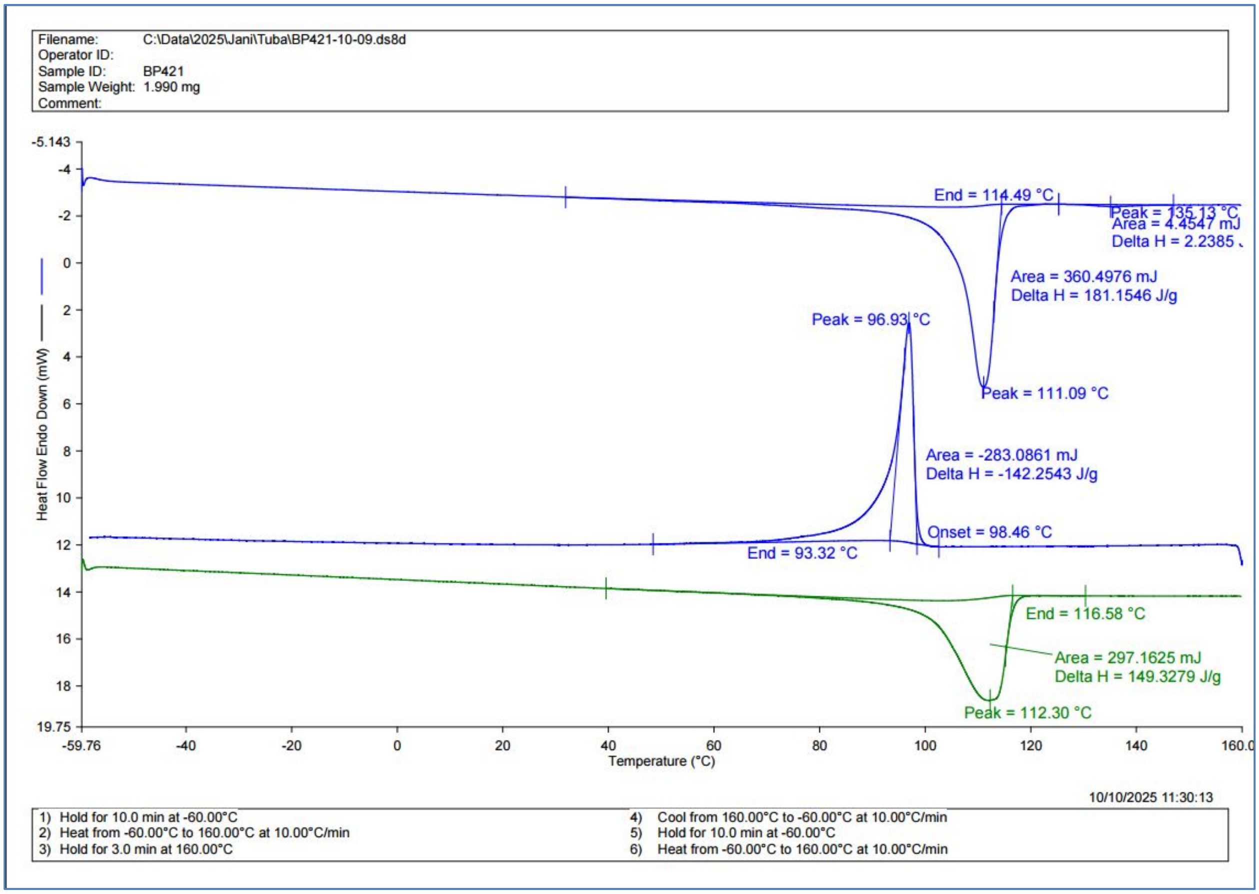

Figure S29. DSC traces of the hydrogenated **ROIMP-*poly*-(DAS-*co*-CP)-200-H$_2$**

11. References

(1) Tae, J.; Yang, Y. K., *Org. Lett.* **2003**, *5* (5), 741–744.

(2) Dagoneau, D.; Xu, Z.; Wang, Q.; Zhu, J., *Angew. Chemie - Int. Ed.* **2016**, *55* (2), 760–763.

(3) Muthusamy, S.; Azhagan, D., *European J. Org. Chem.* **2014**, *2014* (2), 363–370.
